# Genome-Wide Identification of Circular RNAs Potentially Involved in the Biosynthesis of Secondary Metabolites in *Salvia miltiorrhiza*

**DOI:** 10.3389/fgene.2021.645115

**Published:** 2021-11-05

**Authors:** Mei Jiang, Haimei Chen, Qing Du, Liqiang Wang, Xinyue Liu, Chang Liu

**Affiliations:** ^1^ School of Pharmaceutical Sciences, Qilu University of Technology (Shandong Academy of Sciences), Jinan, China; ^2^ Key Laboratory for Applied Technology of Sophisticated Analytical Instruments of Shandong Province, Shandong Analysis and Test Center, Qilu University of Technology (Shandong Academy of Sciences), Jinan, China; ^3^ Key Laboratory of Bioactive Substances and Resource Utilization of Chinese Herbal Medicine from Ministry of Education, Engineering Research Center of Chinese Medicine Resources from Ministry of Education, Institute of Medicinal Plant Development, Chinese Academy of Medical Sciences, Peking Union Medical College, Beijing, China; ^4^ Key Laboratory of Plant Resources of Qinghai-Tibet Plateau in Chemical Research, College of Pharmacy, Qinghai Nationalities University, Xining, China; ^5^ College of Pharmacy, Heze University, Heze, China; ^6^ School of Chinese Materia Medica, Beijing University of Chinese Medicine, Beijing, China

**Keywords:** *Salvia miltiorrhiza*, circular RNAs, SmAACT1, SmDXS2, SmHDR1, SmKSL2, secondary metabolites

## Abstract

Circular RNAs (circRNAs) play various roles in cellular functions. However, no studies have been reported on the potential involvement of circRNAs in the biosynthesis of secondary metabolites in plants. Here, we performed a genome-wide discovery of circRNAs from root, stem and leaf samples of *Salvia miltiorrhiza* using RNA-Seq. We predicted a total of 2,476 circRNAs with at least two junction reads using circRNA_finder and CIRI, of which 2,096, 151 and 229 were exonic, intronic and intergenic circRNAs, respectively. Sequence similarity analysis showed that 294 out of 2,476 circRNAs were conserved amongst multiple plants. Of the 55 predicted circRNAs, 31 (56%) were validated successfully by PCR and Sanger sequencing using convergent and divergent primer pairs. Alternative circularisation analysis showed that most parental genes produced two circRNAs. Functional enrichment analyses of the parental genes showed that the primary metabolism pathways were significantly enriched, particularly the carbon metabolism. Differential expression analysis showed that the expression profiles of circRNAs were tissue-specific. Co-expression analysis showed 275 circRNAs, and their parental genes had significantly positive correlations. However, 14 had significantly negative correlations. Weighted gene co-expression network analysis showed that nine circRNAs were co-expressed with four modules of protein-coding genes. Next, we found 416 exonic circRNAs with miRNA-binding sites, suggesting possible interactions between circRNAs and miRNAs. Lastly, we found six validated circRNAs, namely, SMscf2473-46693-46978, SMscf3091-29256-29724, SMscf16-111773-112193, SMscf432-13232-13866, SMscf7007-10563-10888 and SMscf1730-1749-2013, which were originated from the genes involved in the biosynthesis of secondary metabolites. Their parental genes were acetyl-CoA C-acetyltransferase 1 (SmAACT1), 1-deoxy-d-xylulose-5-phosphate synthase 2 (SmDXS2), 4-hydroxy-3-methylbut-2-enyl diphosphate reductase 1 (SmHDR1), kaurene synthase-like 2 (SmKSL2), DWF4 and CYP88A3, respectively. In particular, the correlation coefficient of SMscf2473-46693-46978 and SmDXS2 gene was 0.86 (*p* = 0.003), indicating a potential interaction between this pair of circRNA and its parent gene. Our results provided the first comprehensive catalogue of circRNAs in *S. miltiorrhiza* and identified one circRNA that might play important roles in the biosynthesis of secondary metabolites.

## Introduction

Circular RNA (circRNA) is a class of non-coding RNA found in eukaryotes and derived from the back-splicing of precursor mRNA (Lasda and Parker, 2014). circRNA was first reported in 1976 (Sanger et al., 1976). In recent years, with the development of high-throughput DNA sequencing technology and bioinformatic analysis methods, circRNAs have been reported in various species, including humans (Memczak et al., 2013), *Archaea* (Danan et al., 2012), *Arabidopsis thaliana* (Ye et al., 2015), rice (Lu et al., 2015) and *Ganoderma lucidum* (Shao et al., 2019). These studies confirmed circRNAs in a wide range of species and revealed their various regulatory functions. CircRNAs can originate from exons (exonic circRNA) (Salzman et al., 2012; Jeck et al., 2013), introns (intronic circRNA) (Zhang et al., 2013), both exons and introns (exon-intronic circRNA) (Salzman et al., 2013) and intergenic regions (intergenic circRNAs) (Zhao W. et al., 2019). The 5 and 3′ ends of circRNAs are linked together to form a covalent closed-loop structure (Chen, 2016). The circularisation of linear RNA molecules enhances the stability of the resulting circRNAs, making them resistant to RNase R that preferentially degrades linear molecules (Jeck et al., 2013).

CircRNAs play important regulatory roles in gene expression regulation (Zhao W. et al., 2019). In mammals, circRNAs can serve as microRNA (miRNA) sponges to bind specific miRNAs, preventing them from regulating target genes (Hansen et al., 2013). In plants, circRNAs can regulate splicing of their parental genes through R-loop formation (Conn et al., 2017). In addition, plant circRNAs play roles in the developmental processes and responses to abiotic and biotic stimuli. For example, in tomatoes, the overexpression of a circRNA originated from phytoene synthase 1 gene can reduce the phytoene synthase 1 mRNA abundance and the accumulation of lycopene β-carotene (Tan et al., 2017). In *Vitis vinifera*, a circRNA (Vv-circATS1) originated from glycerol-3-P acyltransferase was identified, and the overexpression of Vv-circATS1 in *A. thaliana* improved cold tolerance (Gao et al., 2019). In maize, a circRNA (circGORK) originated from Guard cell outward-rectifying K+ -channel gene was identified, and the transgenic line overexpressing circGORK was more tolerant to drought than the control line with higher expression level for several abscisic acid-responsive marker genes, including pyrroline-5-carboxylate synthetase 1, responsive to desiccation 20, responsive to desiccation 22, responsive to desiccation 29A, responsive to desiccation 29B and ABRE binding factor 4 gene (Zhang P. et al., 2019). Moreover, in rice, the transgenic line overexpressing circR5g05160 formed smaller disease lesions and supported significantly less fungal growth of *Magnaporthe oryzae* than the control line, indicating enhanced resistance to the blast disease (Fan et al., 2020). Therefore, circRNAs play broad and important roles in plant development. At present, circRNAs have been identified in about 30 plant species (Zhao X. et al., 2019). However, no reports have focused on the potential roles of circRNAs in the biosynthesis of secondary metabolites.

*Salvia miltiorrhiza* belongs to the family Lamiaceae. It is a widely used medicinal material in East Asia countries (Su et al., 2015). Many diterpenoid secondary metabolites are identified in *S. miltiorrhiza* (Wang et al., 2007). For example, Tanshinones are the primary active compounds of *S. miltiorrhiza*, which have inhibitory activities against calcium channel (Lam et al., 2006) and acetylcholinesterase (Zhou et al., 2011). Gibberellins are a classical phytohormone that plays important roles in plant growth and development (Tyler et al., 2004). Isopentenyl diphosphate (IPP) and its isomer dimethylallyl diphosphate (DMAPP) are the precursors of all terpenoids produced by the methylerythritol phosphate (MEP) pathway (Schwarz, 1994) and mevalonate (MVA) pathway (Rodríguez-Concepción and Boronat, 2002). Most genes in the MEP and MVA pathways have been identified in *S. miltiorrhiza* (Ma et al., 2015), such as 1-deoxy-d-xylulose-5-phosphate synthase 2 (SmDXS2) gene (Zhou et al., 2016), 4-hydroxy-3-methylbut-2-enyl diphosphate reductase 1 (SmHDR1) gene (Hao et al., 2013) and acetyl-CoA C-acetyltransferase 1 (SmAACT1) gene (Cui et al., 2010). Since the report of the whole genome sequence of *S. miltiorrhiza* (Xu et al., 2016), many gene families and transcription factors related to tanshinone biosynthesis have been systematically identified, including the cytochrome P450 family (Chen et al., 2014), 2-oxoglutarate-dependent dioxygenase family (Xu and Song, 2017), basic leucine zipper family (Zhang et al., 2018) and miRNAs (Li et al., 2019). However, circRNAs and their potential roles in the biosynthesis of tanshinone have not been systematically studied in *S. miltiorrhiza*.

Here, we performed RNA sequencing on different tissue samples to identify the circRNAs in *S. miltiorrhiza* for the first time. We described the characteristics of predicted circRNAs and analysed the differential expression of circRNAs amongst the three tissues, the correlation of the expression profiles with their parental genes and the interaction network with miRNA. Notably, we identified and analysed six circRNAs that originated from genes involved in the biosynthesis of secondary metabolites. The results provided a genome-wide catalogue of circRNAs in *S. miltiorrhiza* and the first line of evidence that circRNAs are possibly involved in the expression regulation of genes in the biosynthetic pathways for secondary metabolites.

## Materials and Methods

### Sample Collection

*S. miltiorrhiza* 99-3 line plants were reproduced vegetatively via root and grown under natural conditions in the garden of the Institute of Medicinal Plant Development (Beijing, China, Geospatial coordinate: N40°2′1″, E116°16′5″). Root, stem and leaf samples were collected from three 2-year-old plants and stored immediately in liquid nitrogen at −80°C after collection for long-term storage.

### RNA Extraction, Library Construction and RNA Sequencing

Nine samples from three tissues (leaf, root and stem) with three biological replicates were used to extract total RNA using an RNA extraction kit (TianGen, China). The integrity and quality of the total RNA were evaluated by using the Agilent 2100 Bioanalyzer (Agilent Technologies, United States). The total RNA was detected by using the NanoDrop 2000 spectrophotometer (Thermo Scientific, United States). Five micrograms of RNA per sample was used as input material for RNA sample preparations. Firstly, ribosomal RNA was removed by using the Epicentre Ribo-zero™ rRNA Removal Kit (Epicentre, United States), and the rRNA-free residue was cleaned up by ethanol precipitation. Subsequently, the linear RNA was digested with 3 U of RNase R (Epicentre, United States) per microgram of RNA in accordance with previous studies (Zhao W. et al., 2017; Ye et al., 2017). According to the manufacturer’s instructions, the RNase R-treated RNA was used to construct libraries using the NEBNext Ultra™ Directional RNA Library Prep Kit. The libraries were sequenced using a Hiseq 4000 platform (Illumina, United States) with 2 × 150 bp paired-end reads. Clean data were obtained by removing low-quality sequences using the FASTX Toolkit (v0.0.13): the percentage of bases with a quality value of *Q* < 20 was more than 50%, and the percentage of ‘N’ was more than 5%. The raw data were deposited into the NCBI SRA database with the following accession number: SRR11126657, SRR11126658 and SRR11126659.

### Prediction of CircRNAs

Two software tools, circRNA_finder (v2.6.1d) (Westholm et al., 2014) and CIRI (v2.0.6) (Gao et al., 2015), were used to predict circRNAs on the basis of the genome sequence of *S. miltiorrhiza* as they could lower the false positive rate. We used the genome sequence of *S. miltiorrhiza* 99-3 line (Xu et al., 2016) as the reference sequence to improve the accuracy of circRNA identification, which is the same line as that used in this project. For circRNA_finder (v2.6.1d), we used STAR (v2.6.0a) (Dobin et al., 2012) to align the clean reads to the reference genome with default options: STAR--runMode genomeGenerate--genomeDir STAR_index--genomeFastaFiles genome.fasta--sjdbGTFfile genome.gff; perl runStar.pl -inFile1 R1.fq.gz -inFile2 R2.fq.gz--genomeDir STAR_index–outPrefix CIRC_Prefix. Then, we used the postProcessStarAlignment.pl module to find circRNAs with default options: perl postProcessStarAlignment.pl--starDir CIRC_Prefix. For CIRI (v2.0.6), we used BWA (v0.7.12) (Vasimuddin et al., 2019) to align the clean reads to the reference genome with default options: bwa mem genome.fasta R1.fq.gz R1.fq.gz > CIRC.sam. We used the CIRI2.pl module to find circRNAs with default options: perl CIRI2.pl -I CIRC.sam -O outfile -F genome.fasta -A genome.gff.

The analysis pipeline was divided into three steps. In step 1, we predicted the circRNAs in each of the nine samples (L_R1, L_R2, L_R3, R_R1, R_R2, R_R3, S_R1, S_R2 and S_R3, L: leaf, R: root; S: stem; R: replicate) by two software tools: circRNA_finder (v2.6.1d) (Chen et al., 2016) and CIRI (v2.0.6) (Gao et al., 2015). The circRNAs with junction reads ≥2 were retained as described previously (Ye et al., 2015; Tong et al., 2018; Zhang X. et al., 2019). In step 2, we compared the circRNAs predicted by the two programs. Firstly, we combined all the circRNAs predicted by both software tools to form a union set for each sample. Then, we selected the circRNAs predicted by both software tools to form an intersection set. The ratio of the numbers in the intersection set and union set was calculated. In step 3, we combined the circRNAs predicted by both software tools amongst all nine samples.

### Conservation of CircRNAs

We searched the PlantcircBase (Chu et al., 2017) and PlantCircNet (Zhang et al., 2017) databases for homologs using BLASTN using the following parameters to determine the conservation of circRNAs identified in *S. miltiorrhiza*: a word size of 11 and an e value cutoff of 1E^−5^.

### Validation of CircRNAs

We selected 55 circRNAs to validate the predicted circRNAs in *S. miltiorrhiza*. We designed convergent and divergent primers to amplify genomic DNA (gDNA) and complementary DNA (cDNA). The convergent primers would generate products from gDNA and cDNA, whereas the divergent primers would generate products only from cDNA. Next, the PCR products with the expected size were further sequenced using the Sanger method.

Total DNA and RNA were extracted using an extraction kit (TianGen, China). We incubated the total RNA for 15 min at 37°C with 3 units/µg of RNase R (Epicentre, United States) to remove the linear RNA. The RNase R-treated RNA was then reverse transcribed to cDNA with random primers using a reverse transcription kit (TransGen Biotech, China). Approximately 20 ng of total DNA or cDNA, 10 μM of convergent or divergent primers, Taq DNA polymerase and 10× buffer were used for PCR amplification under the following conditions: 95°C for 2 min; 40 cycles of 94°C for 30 s, 60°C for 30 s and 72°C for 30 min and 72°C for 5 min.

### Differential Expression Analysis of CircRNAs

We used the number of junction reads to quantify the expression of circRNAs across the three tissues. The expression level was represented by Reads of exon model Per Million mapped reads (RPM), calculated by the total number of junction reads divided by the total number of sequencing reads in the corresponding sample. The average expression level of three biological replicates was used to calculate the corresponding fold change value. We calculated the false discovery rate (q value) using R’s fdrtool package (v3.60).

### Correlation of the Expression Profiles of Exonic CircRNAs and Their Parental Genes

We compared the expression profiles of 2,247 circRNAs and their parental genes to determine potential interactions between the circRNAs and their parental genes. We assembled the clean reads to the transcripts using cufflinks (v2.2.1) to obtain the transcripts of parental genes (Trapnell et al., 2010). The transcripts located in the same genomic position as the circRNA was considered as the transcript of the parental genes was used to calculate the Fragment Per Kilobase of exon per Million fragments mapped (FPKM) of parental genes. RPM and FPKM represented the expression levels of exonic circRNAs and parental genes, respectively. Then, we calculated the Pearson correlation coefficient of the expression of circRNAs and their parental genes using Python’s Pearson function (2.7.12).

### Correlation of the Expression Profiles of Exonic CircRNAs and Protein-Coding Genes

Based on the annotation of the reference genome (Xu et al., 2016), we extracted the assembled transcripts belonging to the protein-coding genes. Their expression levels were represented by FPKM, which was calculated by using cuffdiff (Trapnell et al., 2010). Then, we used the WGCNA package to identify the co-expression modules using the following parameters: power = 8, maxBlockSize = 30000, minModuleSize = 30 (Langfelder and Horvath, 2008). Finally, we analysed the correlation of expression profiles of each module and exonic circRNAs using the WGCNA package (Langfelder and Horvath, 2008). The genes in the module, which were significantly correlated with circRNAs, were subjected to Kyoto Encyclopedia of Genes and Genomes (KEGG) analysis by DAVID web server (Huang et al., 2009).

### Identification of miRNA Targeting CircRNAs and mRNAs for Cleavage

The psRNATarget web server was used to identify the circRNAs with miRNA-binding sites using the default parameters of Schema V2 (Dai et al., 2018). The miRNA sequences of *S. miltiorrhiza* used in this study were reported in a previous study (Xu et al., 2014). The parental sequences of circRNAs and mRNA were used as the targeted sequences.

### Functional Enrichment Analysis of Parent Genes

We performed GO term and KEGG pathway enrichment analyses of the parental genes with exonic and intronic circRNAs to identify their potential functions. The sequences of parental genes were extracted by the bedtools program (v2.25.0) and then annotated by the DAVID web server (Huang et al., 2009). All genes in the genome of *S. miltiorrhiza* were used as background. Then, we extracted the results related to Gene Ontology (GO) and KEGG. The GO term and KEGG pathway with a *q*-value < 0.05 and a minimum gene count of 3 were considered as significantly enriched.

## Results

### Identification of CircRNAs in *S. miltiorrhiza*

RNA samples were extracted from three types of tissues, including root, stem and leaf, with three biological replicates from each tissue. In total, average 75,297,184 raw reads per sample were generated, and average 57,007,959 clean reads per sample were obtained after trimming the adapter sequences and removing low-quality reads (Supplementary Table S1).

We obtained average 645, 1,405 and 1,276 circRNAs with at least two junction reads from the leaf, root and stem tissue samples by using circRNA_finder software, respectively. By contrast, we obtained 684, 1,342 and 1,251 circRNAs with at least two junction reads from the leaf, root and stem tissue samples by using CIRI software, respectively. The results identified by both software tools obtained average 42.14, 44.72 and 40.06% circRNAs from the leaf, root and stem tissue samples, respectively. In total, 2,476 circRNAs were identified after removing duplicate circRNAs amongst the leaf, root and stem tissues (Supplementary Table S2).

**FIGURE 1:**
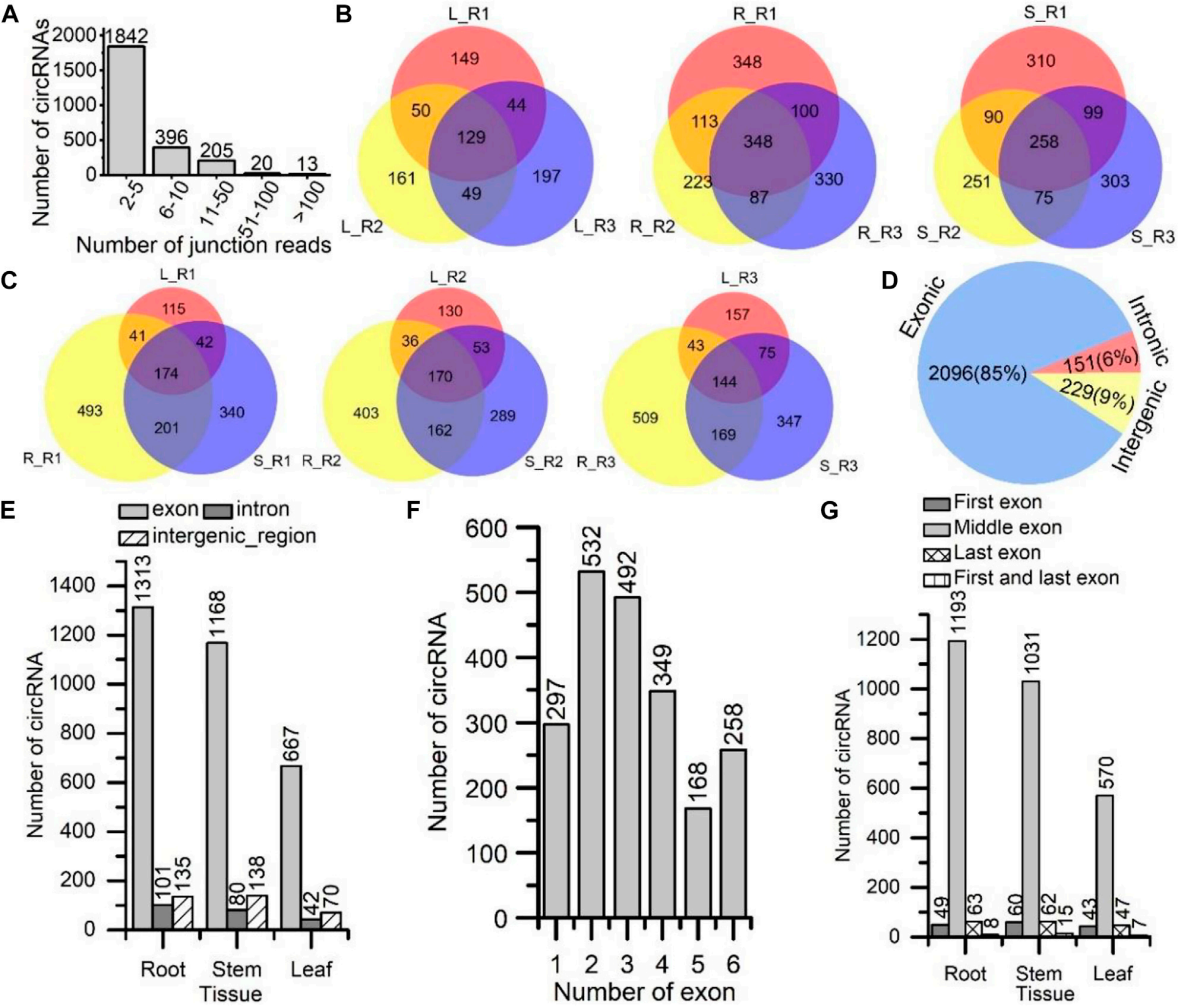
Identification and characterization of circRNAs in *S. miltiorrhiza*. **(A)** The number of circRNAs categorized by the number of associated junction reads. The X-axis shows the number of junction reads. The Y-axis shows the number of circRNAs. **(B)** Venn diagrams comparing the circRNAs identified among three plant replicate samples for the same tissue type. L, leaf; R, root; S, stem; R1, replicate sample from plant No. 1 (red); R2, replicate sample from plant No. 2 (yellow); R3, replicate sample from plant No. 3 (blue). **(C)** Venn diagrams comparing the circRNAs identified among three types of tissues for the same plant individuals (No. 1, 2, and 3), leaf (L: red), root (R: yellow), and stem (S: blue). **(D)** Numbers of circRNAs are categorized by origin types: exon, intron, and intergenic region, across all three tissues. **(E)** Numbers of circRNAs are categorized by the types of their origins: exon, intron, and intergenic region, in each tissue. The X-axis shows the tissue type. **(F)** The numbers of circRNAs are categorized by the numbers of back-splicing exons that the circRNA had spanned. The X-axis shows the numbers of exons. **(G)** The numbers of circRNAs are categorized by their origins: first exon, middle exon, last exon, first, and last exon. The X-axis shows the tissue type. In **A, E, F, and G**, the Y-axis is the number of circRNAs. The number on the top of each bar represents the number of circRNAs.

The circRNAs having, on average, 2–5 junction reads were the most abundant, followed by those having 6–10 and 11–50 junction reads (Figure 1A). Then, we compared these 2,476 circRNAs at the intra- and inter-tissue levels. For the intra-tissue comparison, 129 of 779 (16.56%), 348 of 1,549 (22.47%) and 258 of 1,386 (18.61%) circRNAs were shared amongst the leaf, root and stem tissue samples, respectively (Figure 1B). By contrast, for inter-tissue analysis, 174 out of 1,406 (12.38%), 170 out of 1,243 (13.68%) and 144 out of 1,444 (9.97%) circRNAs were shared amongst the three paired tissues (Figure 1C). These results indicated a high degree of variations amongst the circRNAs identified at the intra-tissue and inter-tissue levels.

### Features of CircRNAs in *S. miltiorrhiza*

On the basis of junction site’s genomic location, we classified these circRNAs into three types: exonic, intronic and intergenic circRNAs. A total of 2,096 exonic, 151 intronic and 229 intergenic circRNAs were found across all three tissues (Figure 1D). Exonic circRNAs were the most abundant. Notably, for exonic circRNAs, the corresponding genes were their parent genes. In each tissue, the number of exonic circRNA was significantly higher than those of intronic and intergenic circRNAs (Figure 1E). The results showed that circRNAs could originate from diverse genomic locations, particularly from the exonic regions.

We also calculated the number of exons spanned by the exonic circRNAs (Figure 1F). Of the 2,096 (25.38%) circRNAs, 532 spanned two exons, which was the most abundant type. Of the 2,096 (23.47%) circRNAs, 492 spanned three exons, which was also abundant. The distribution of spanned exons was consistent with those in *A. thaliana* (Ye et al., 2015) and *Arachis hypogaea* (Zhang X. et al., 2019). Next, we analysed the genomic position of the exonic circRNAs and divided them into four groups: circRNAs were mapped to the first exon, middle exon, last exon and both the first and last exons (Figure 1G). Most circRNAs (570–1193) originated from the middle exons in all three tissues. 47–63 and 43–60 circRNAs originated from the last exon and first exon, respectively. Only a few circRNAs (7–15) derived from the first and last exon.

### Conservation of CircRNAs Identified in *S. miltiorrhiza*

Sequence conservation analysis showed that 294 circRNAs were conserved amongst *Arabidopsis thaliana*, *Cucumis sativus*, *Camellia sinensis*, *Gossypium arboretum*, *Gossypium hirsutum*, *Glycine max*, *Gossypium raimondii*, *Nicotiana benthamiana*, *Oryza sativa*, *Oryza sativa* ssp. indica, *Pyrus betulifolia*, *Poncirus trifoliata*, *Solanum lycopersicum*, *Solanum tuberosum*, *Brachypodium distachyon*, *Hordeum vulgare*, *Triticum aestivum* and *Zea mays* (Supplementary Table S2). Among them, 82 circRNAs had homologs in both databases.

Some circRNAs were conserved across multiple plant species. For example, SMscf16-111773-112193 originated from the SmAACT1 gene was found in four species (*Arabidopsis thaliana*, *Glycine max*, *Cucumis sativus* and *Oryza sativa* ssp. indica), and SMscf3091-29256-29724 originated from the SmHDR1 gene was found in three species (*Arabidopsis thaliana*, *Camellia sinensis* and *Poncirus trifoliata*).

### Validation of the Predicted CircRNAs in *S. miltiorrhiza*

**FIGURE 2:**
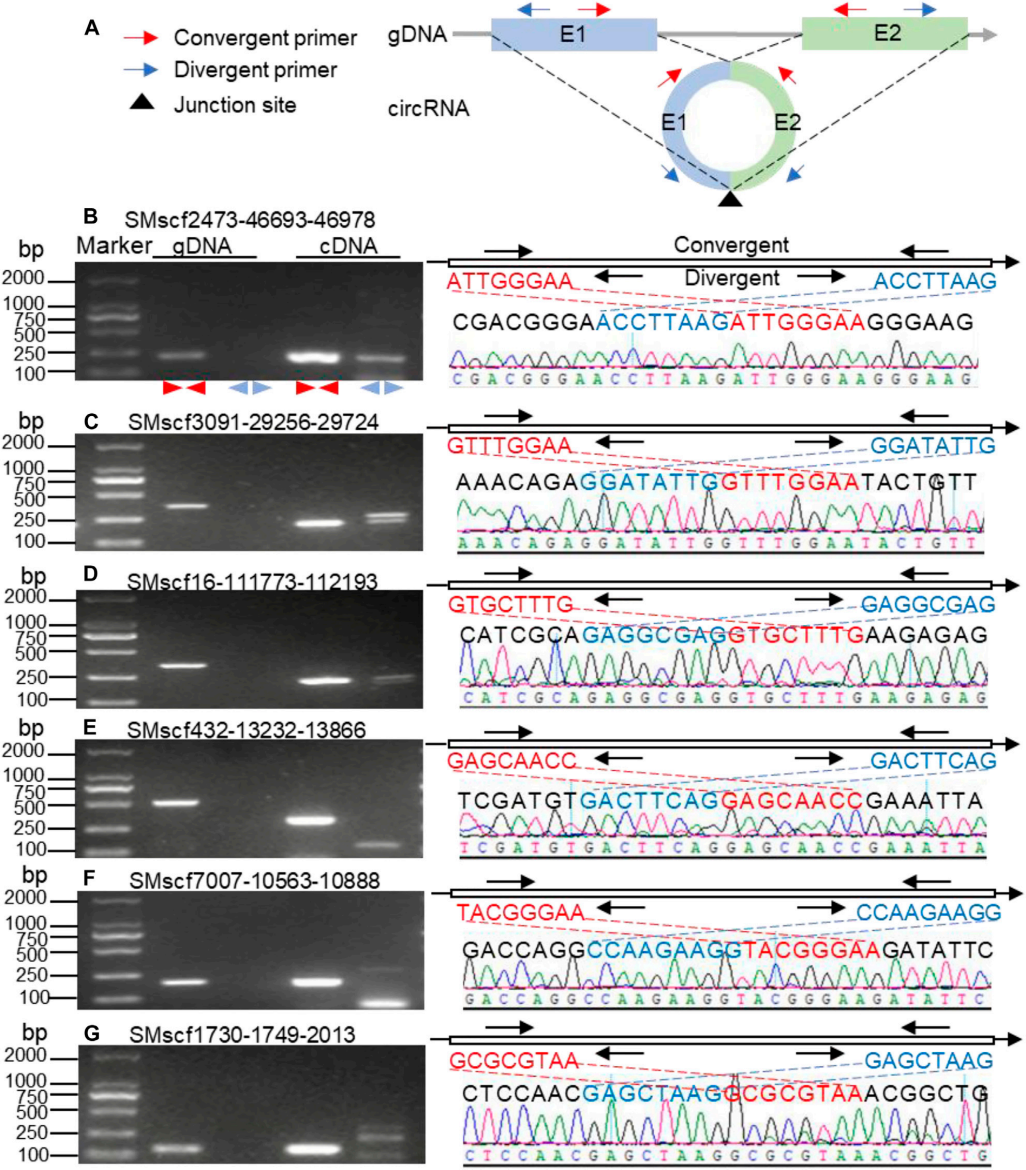
Validation of circRNAs identified in *S. miltiorrhiza*. **(A)** Schematic representation of the validation strategy. Colored rectangles and gray lines represent exons and introns, respectively. E1, exon 1, E2, exon 2. The red and blue arrows represent convergent and divergent primers, respectively. The black triangle under the circle indicates the junction site for circularization. The dashed lines show the position of the junction sites on the genomic DNA. As demonstrated in the figure, both convergent primer pair (red) and divergent primer pair (blue) are expected to produce PCR products using the cDNA of the circRNA as a template. In contrast, only the convergent primer pair is expected to produce PCR products using genomic DNA as a template. However, due to the presence of the intron, the PCR products of the circRNA and genomic DNA are different for the same convergent primer pair. **(B-G)** the electrophoretic gel picture of the PCR results. The type of template for PCR is shown on the top of each gel picture. The type of primer is shown at the bottom of each gel. gDNA, genomic DNA; cDNA, complementary DNA; “
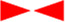
”, convergent primer pair; “
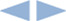
”, divergent primer pair. The right part in each panel compares the sequences around the junction sites with those obtained from Sanger sequencing. The upper right part in each panel shows the schematic representation of the sequences around the junction sites of the circRNAs. The thin line represents the genome sequence. The red sequence locates downstream of the junction site, while the blue sequence locates upstream of the junction site. The convergent and divergent primer pairs are shown above and below the genomic sequence, respectively. The mapping of the sequences around the expected junction sites and the sequences obtained from Sanger sequencing are connected with dashed lines.

We selected 55 circRNAs for further validation if they meet one of the two conditions: 1) the target genes of these circRNAs were involved in the biosynthesis of bioactive compounds; 2) the circRNAs were the most abundant based on the number of the corresponding junction reads. Of the 55 (56%) circRNAs, 31 was amplified successfully (Figure 2 and Supplementary Figure S2). The sequencing results of the 31 amplified products were consistent with the expected back-splice sites. The sequences of 31 validated circRNAs are shown in the Supplementary Material S3 file. The results of Sanger sequencing are shown in the Supplementary Material S4 folder. The success rate for validation was comparable to those described for other plants (Ye et al., 2015; Zeng et al., 2018).

### Alternative Circularisation of CircRNAs in *S. miltiorrhiza*

We analysed the alternative circularisation of 2,096 exonic circRNAs. Most parental genes (1,168 out of 2,096) produced only one circRNA (Supplementary Figure S1). The other circRNAs were produced from 420 distinct parent genes. Of the 420 (63.57%) parental genes, 267 produced two circRNAs, and this was the most abundant type of alternative circularisation event. In addition, 102 out of 420 (24.29%) parent genes had three circRNAs each. The most complex gene was SMil_00000685, which produced 11 circRNAs. A schematic representation of the alternative circularisation events for this gene is shown in Supplementary Figure S3.

**FIGURE 3:**
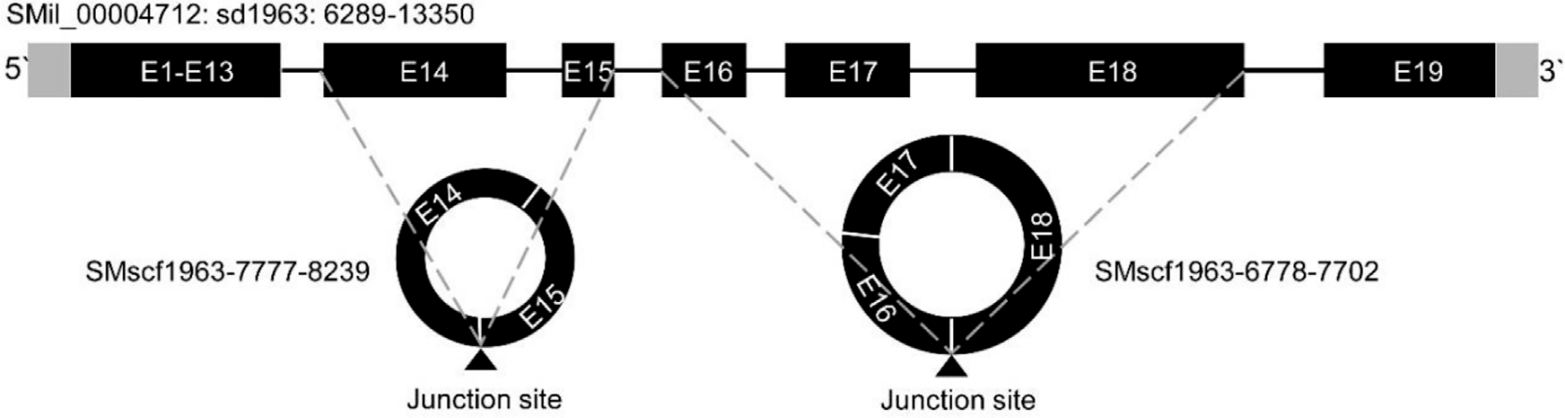
Schematic representation of the alternative circularization of gene SMil_00004712 in *S. miltiorrhiza*. The structure of the parent genes is based on that described previously (Xu et al., 2016). The gray and black rectangles represent untranslated regions (UTR) and exons, respectively. The thin lines between the exons represent introns. The two circles represent two circRNAs SMscf 1963-7777-8239, and SMscf 1963-6778-7702 derived the gene. The exons are labeled with “E” followed by the number of the exons. The black triangles under the circles indicate the junction sites. The dashed lines indicate the positions of the junction sites on the genomic sequence.

Most of the alternative circularisation events were based on the bioinformatic analysis. However, we validated one alternative circularisation event. The SMil_00004712 gene could produce two circRNAs, namely, SMscf 1963-6778-7702 and SMscf 1963-7777-8239. The schematic relationship of the parent gene and two circRNAs is shown in Figure 3. SMscf 1963-7777-8239 originated from the circularisation of exon14 and exon15. SMscf 1963-6778-7702 originated from the circularisation of exon16 and exon18. PCR amplification of gDNA and cDNA with the convergent and divergent primers confirmed the circular form of the two circRNAs (Supplementary Figures S2E,F). Mapping of the sequencing results of PCR products confirmed the circularisation of the exons (Supplementary Figures S2E,F).

### Functional Enrichment Analyses of CircRNAs’ Parental Genes

**FIGURE 4:**
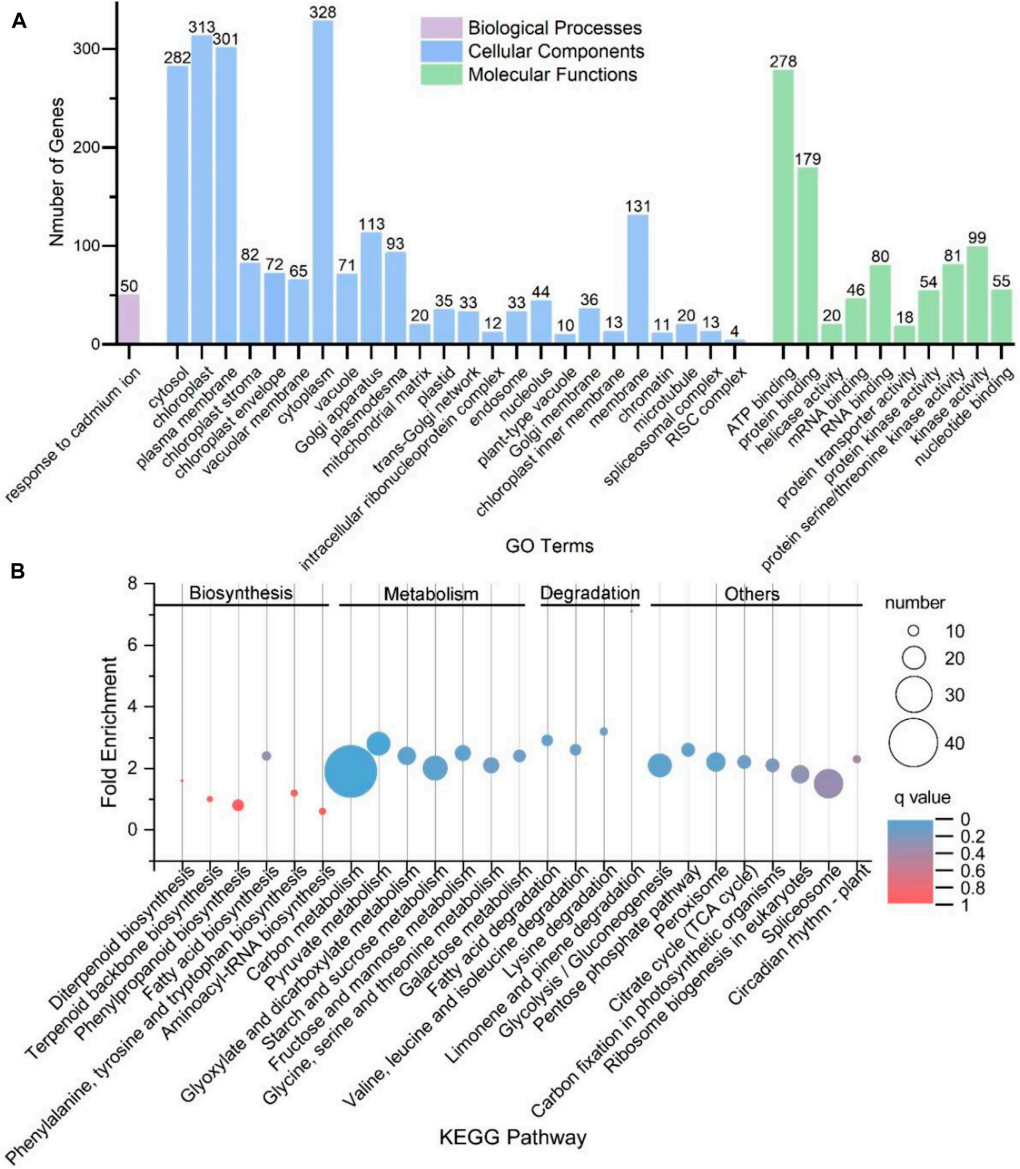
Functional enrichment analyses for parental genes of circRNAs in *S. miltiorrhiza*. **(A)** Thirty-five significantly enriched GO terms for the parental genes of circRNAs. The X-axis shows the names of the GO terms. The Y-axis shows the number of genes belonging to each GO term. These terms are classified into three categories: Biological Processes (purple), Cellular Components (blue), and Molecular Functions (green). The terms with a *q*-value < 0.05 and a minimum gene count of 3 were considered significantly enriched. **(B)** KEGG pathways for the parental genes of circRNAs with a minimum gene count of 3. The X-axis shows the name of the pathways. The Y-axis shows the “Fold Enrichment.” The pathways are divided into four categories: Biosynthesis, Metabolism, Degradation, and others, which are shown above the figure. The size of the circle correlates with the number of genes. The color of the circle indicates different q values.

Functional enrichment analyses showed that these genes were mapped to a broad range of GO terms, including 106 subgroups under the category of biological processes, 58 subgroups under the category of cellular components and 56 subgroups under the category of molecular functions (Supplementary Table S4). A total of 35 GO terms were significantly enriched, including one subgroup of biological processes, 24 subgroups of cellular components and ten subgroups of molecular functions (Figure 4A). Moreover, these genes were mapped to 25 KEGG pathways, including 6, 7 and 4 pathways, which were related to biosynthesis, metabolism and degradation pathways (Figure 4B). Among them, five KEGG pathways were significantly enriched, namely, carbon metabolism; pyruvate metabolism; biosynthesis of antibiotics; valine, leucine and isoleucine degradation and lysine degradation.

### Differential Expression of CircRNAs Amongst the Three Tissues in *S. miltiorrhiza*

**FIGURE 5:**
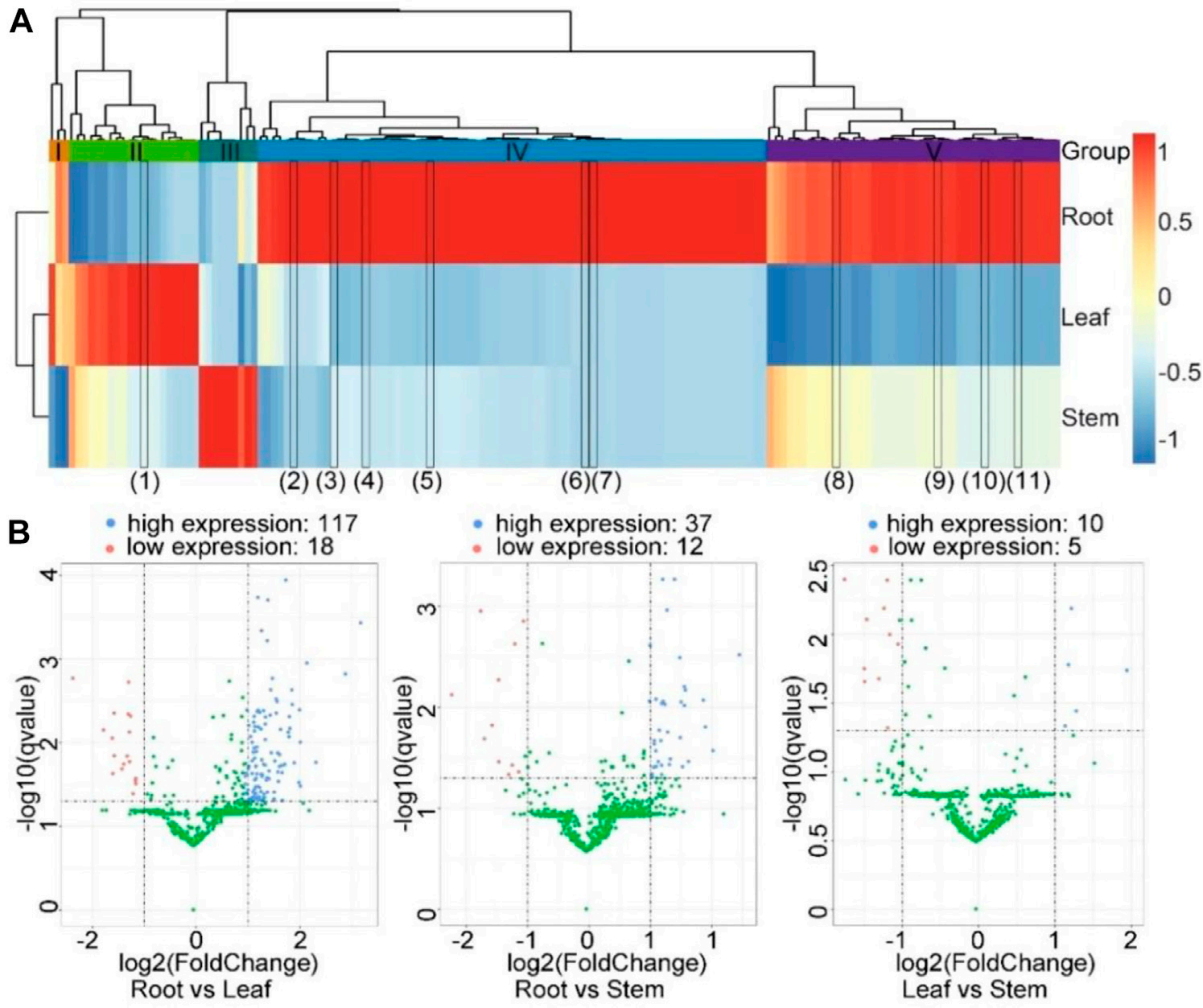
Differential expression of circRNAs among the three tissues. **(A)** The heatmap showed the expression profiles of the circRNAs across three tissues. The color corresponds to the Z-score transformed from the RPM values of circRNAs in the corresponding tissue types. The color panel is shown on the right. The tissues were clustered in the horizontal direction, while the circRNAs were clustered in the vertical direction. Five clusters of circRNAs, I, II, III, IV, and V, are shown between the hierarchical tree and heatmap. The black rectangles in the heatmap represent the validated circRNAs that were differentially expressed among the three tissues, from left to right: (1) SMscf5077-22608-23239, (2) SMscf2473-46693-46978, (3) SMscf4140-50187-50921, (4) SMscf1368-52664-53164, (5) SMscf16-111773-112193, (6) SMscf550-26951-27637, (7) SMscf1271-41093-41414, (8) SMscf2943-49422-49697, (9) SMscf2773-28597-28968, (10) SMscf354-48544-48880, (11) SMscf534-16776-17238. **(B)** The volcano plots showing the differential expressions of circRNAs between the root and leaf, root and stem, and leaf and stem samples. The X-axis is the log2(fold change), and the Y-axis is the −log_10_ (*q* value). The horizontal dashed line indicates where the *q* value equals 0.05, and the vertical dashed line indicates where the fold change equals 1 or −1, respectively. Those with a q value <0.05 and log2(fold change) ≥ 1 were considered significantly highly expressed and represented by blue points. In contrast, those with a *q* value <0.05 and log2(fold change) ≤ −1 were considered significantly lowly expressed and represented by red points. The rest of the circRNAs were considered less significantly differentially expressed and represented by green points. The numbers of significantly highly and lowly expressed genes in each comparison are shown above the corresponding volcano plots.

A total of 155 significantly differentially expressed circRNAs were identified under two conditions: |log2(fold change) | ≥ 1 and *q* ≤ 0.05. These circRNAs included 134 exonic circRNAs and 21 intronic circRNAs (Supplementary Table S5). As shown in the horizontal direction of the heatmap, the expression profiles of circRNAs from the leaf and stem were similar to those of the root (Figure 5A). From the perpendicular direction, the genes were clustered into five clusters (I to V). CircRNAs from cluster I were expressed the highest in the root. CircRNAs from cluster II were expressed the highest in the leaf, although the relative expression levels in the leaf *vs* those in the other two tissues varied. CircRNAs from cluster III were expressed the highest in the stem. CircRNAs from clusters IV and V were expressed the highest in the root. However, the expression levels of circRNAs from cluster IV were comparable in the leaf and stem tissues. By contrast, the expression levels of circRNAs from cluster V in the leaf were significantly lower than those in the stem. These distinct expression profiles indicated that these circRNAs might play different functions across the three tissue types.

Next, we used the volcano plot to visualise the genes on the basis of the corresponding fold changes and q values. In particular, the expression levels of 117 and 18 circRNAs in the root were significantly higher and lower than those in the leaf, respectively. The expression levels of 37 and 12 circRNAs in the root were significantly higher and lower than those in the stem, respectively. Moreover, the expression levels of 5 and 10 circRNAs in the leaf were significantly higher and lower than those in the stem, respectively (Figure 5B).

### Co-Expression of Exonic CircRNAs and Their Parental Genes in *S. miltiorrhiza*

The expression profiles of 275 circRNAs and their parent genes were significantly and positively correlated (*r* ≥ 0.5, *p* < 0.05). Among them, 45 pairs were highly correlated (*r* ≥ 0.9, *p* < 0.05). By contrast, 14 circRNAs and their parent genes were significantly and negatively correlated (*r* ≤ −0.5, *p* < 0.05, Supplementary Table S6).

### Identification of miRNA-Targeting CircRNAs and mRNAs for Cleavage in *S. miltiorrhiza*

CircRNAs could regulate the expression level of miRNA target genes by binding to miRNAs and preventing them from degrading the target genes (Hansen et al., 2013). We identified the circRNAs with miRNA-binding sites using the psRNATarget webserver to explore the potential interactions between exonic circRNAs and miRNAs in *S. miltiorrhiza*. A total of 416 circRNAs had miRNA-binding sites, which met the conditions of reverse complementarity matching with circRNAs and the target-site accessibility evaluation. The matching information of circRNAs and miRNAs is listed in Supplementary Table S7. Multiple relationships were found between miRNA and circRNAs. For example, miR395a was predicted to have binding sites amongst 13 circRNAs, having the largest number of targeting circRNAs (SMscf1280-66530-68013, SMscf1233-21627-22439, SMscf3102-30466-34020, SMscf367-74519-75487, SMscf367-74519-75634, SMscf53-94130-94864, SMscf7291-3708-5612, SMscf131-128181-129800, SMscf1393-16913-20300, SMscf209-75430-76170, SMscf368-32768-34300, SMscf4608-20154-21285 and SMscf81-73757–75549). However, the identification of miRNA targeting was based on computational predictions. The reliability of these predictions needed further investigation.

### Functional Annotation, Differential Expression and Co-Expression of 31 Validated CircRNAs and Their Parental Genes

**FIGURE 6:**
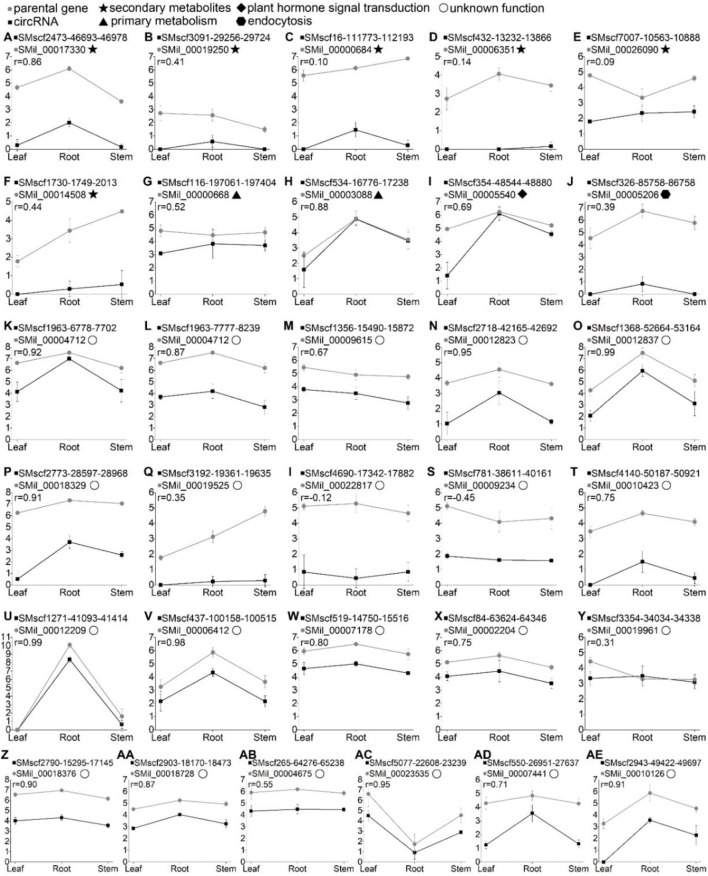
The expression correlation of 31 validated circRNAs and their parental genes across the leaf, root, and stem tissues of *S. miltiorrhiza*. The X-axis shows the three tissues. Y-axis shows expression levels of the circRNAs in log_2_ (RPM) and the parental genes in log_2_ (FPKM). Error bars represent the standard error among three replicates. “
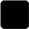
” indicate the circRNA name. “
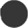
” indicates the parental gene name. “r” represents the correlation coefficient of expression profiles of the circRNAs and their parental genes. The different symbols after the parental gene names represent the functions of parental genes based on the KEGG pathway analyses. “
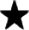
”, the pathway of biosynthesis of secondary metabolites; “
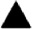
”, the pathway of primary metabolism; “
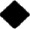
”, the pathway of Plant hormone signal transduction; “
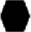
”, the pathway of Endocytosis; “
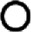
”, the functions of parental genes were unknown.

The parental genes of the 31 validated circRNA were subjected to KEGG pathway analyses. Based on the result, these circRNAs can be divided into five groups. The parental genes of the first group of circRNAs were mapped to the metabolic pathways. Six circRNAs (Figure 2) belonged to this group. Among them, circRNA SMscf2473-46693-46978 had a significantly positive correlation with its parental gene (r = 0.86, *p* < 0.05). The parental genes of group two were mapped to the primary metabolism pathway. Two circRNAs (Supplementary Figures S2A,B) belonged to this group. The parental gene of circRNA SMscf116-197061-197404 was mapped to the glycerolipid metabolism pathway, and another parental gene of circRNA SMscf534-16776-17238 was mapped to the pantothenate and CoA biosynthetic pathways. The correlation coefficient of the two circRNAs and their parental genes was 0.52 (*p* > 0.05) and 0.88 (*p* < 0.05; Figures 6G,H). The parental genes of group three were mapped to the plant hormone signal transduction pathway. Only circRNA SMscf354-48544-48880 (Suppelementary Figure S2C) was included in this group, and the correlation coefficient of this circRNA and its parental gene was 0.69 (*p* < 0.05, Figure 6I). The parental gene of group four was mapped to the endocytic pathway. Only circRNA SMscf326-85758-86758 (Supplementary Figure S2D) was included in this group, and the correlation coefficient of this circRNAs and its parental gene was 0.39 (*p* > 0.05, Figure 6J). The functions of the parental genes of group five were unknown. A total of 21 circRNAs (Supplementary Figures S2E–Y) were included in this group. Among them, 16 circRNAs had a significantly positive correlation with their parental gene (r ≥ 0.5, *p* < 0.05; Figures 6K–Z, AA,AC,AD and AE).

Differential expression analysis showed that 11 out of 31 circRNAs were differentially expressed amongst three tissues. They belonged to three clusters, which were defined on the basis of cluster analysis of differentially expressed circRNAs (Figure 5A). In particular, the circRNA SMscf5077-22608-23239 belonged to cluster II; circRNAs SMscf2473-46693-46978, SMscf4140-50187-50921, SMscf1368-52664-53164, SMscf16-111773-112193, SMscf550-26951-27637 and SMscf1271-41093-41414 belonged to cluster IV; circRNAs SMscf2943-49422-49697, SMscf2773-28597-28968, SMscf354-48544-48880 and SMscf534-16776-17238 belonged to cluster V.

### Co-Expression of 31 Validated CircRNAs and Protein-Coding Genes

**FIGURE 7:**
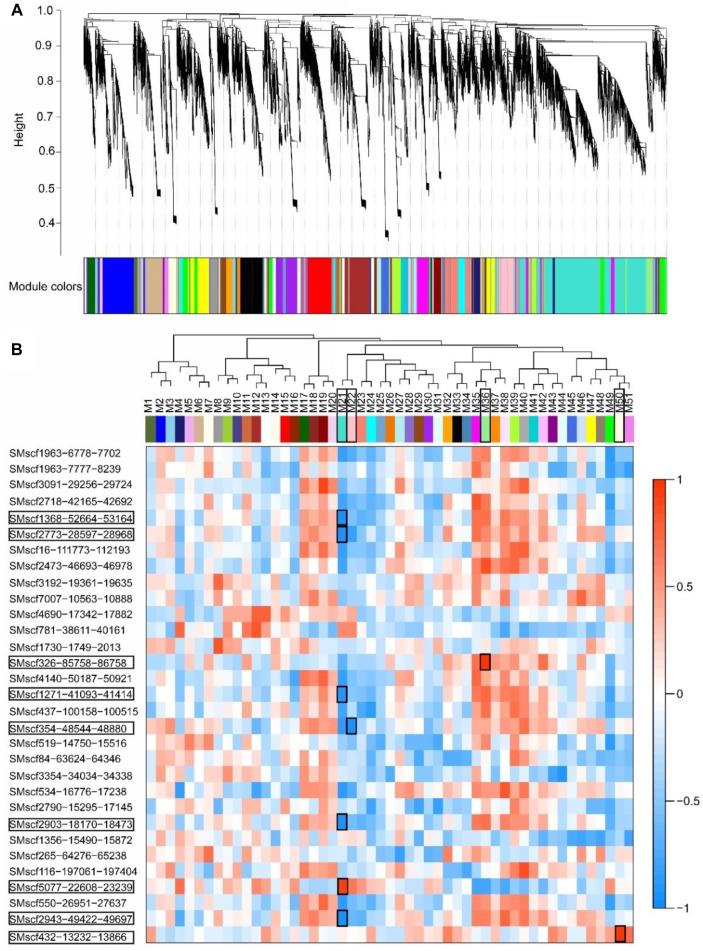
The Co-expression analysis of 31 validated circRNAs and protein-coding genes by WGCNA method. **(A)** Hierarchical cluster dendrogram of modules of protein-coding genes. Modules corresponding to branches were color-coded. **(B)** The correlation of expression profiles of each module and 31 validated circRNAs. The tree showed the relationship between the modules. The heatmap showed the correlation of expression profiles of each module and 31 validated circRNAs. The significantly correlated circRNAs and modules were marked with black rectangles.

A total of 22,978 genes with FPKM more than one were used for WGCNA. These genes were clustered into 51 modules (Figure 7A). The expression profile of four modules, namely, Module21, Module22, Module36 and Module50, had a significant correlation with circRNAs (r ≥ 0.9, *p* < 0.05; Figure 7B). Module21 had a significantly positive correlation with circRNA SMscf5077-22608-23239 (r ≥ 0.9, *p* < 0.05) and a significantly negative correlation with circRNAs SMscf1368-52664-53164, SMscf2773-28597-28968, SMscf1271-41093-41414, SMscf2903-18170-18473 and SMscf2943-49422-49697 (r ≤ −0.9, *p* < 0.05). Module22 had a significantly negative correlation with SMscf354-48544-48880 (r ≤ −0.9, *p* < 0.05). Module36 had a significantly positive correlation with SMscf326-85758-86758 (r ≥ 0.9, *p* < 0.05). Module50 had a significantly positive correlation with SMscf432-13232-13866 (r ≥ 0.9, *p* < 0.05).

Furthermore, the genes in the four modules were subjected to KEGG enrichment analyses. Module21 contained 3,463 genes, showing significant enrichment of genes from the biosynthesis of secondary metabolites, including the stilbenoid, diarylheptanoid and gingerol biosynthetic pathways; zeatin biosynthetic pathway and flavonoid biosynthetic pathway. Module22 contained 704 genes, showing no significant enrichments of genes in any pathways. By contrast, Module36 contained 275 genes, showing significant enrichment of genes from the nicotinate and nicotinamide metabolism pathways. Module50 had 270 genes, showing significant enrichment of genes from the flavonoid biosynthetic pathway. Nine circRNAs were co-expressed with four modules, which indicated that these circRNAs may regulate gene expressions, particularly for genes involved in the biosynthesis of secondary metabolites.

### CircRNAs Originated From the Genes Involved in the Biosynthesis of Secondary Metabolites

**FIGURE 8:**
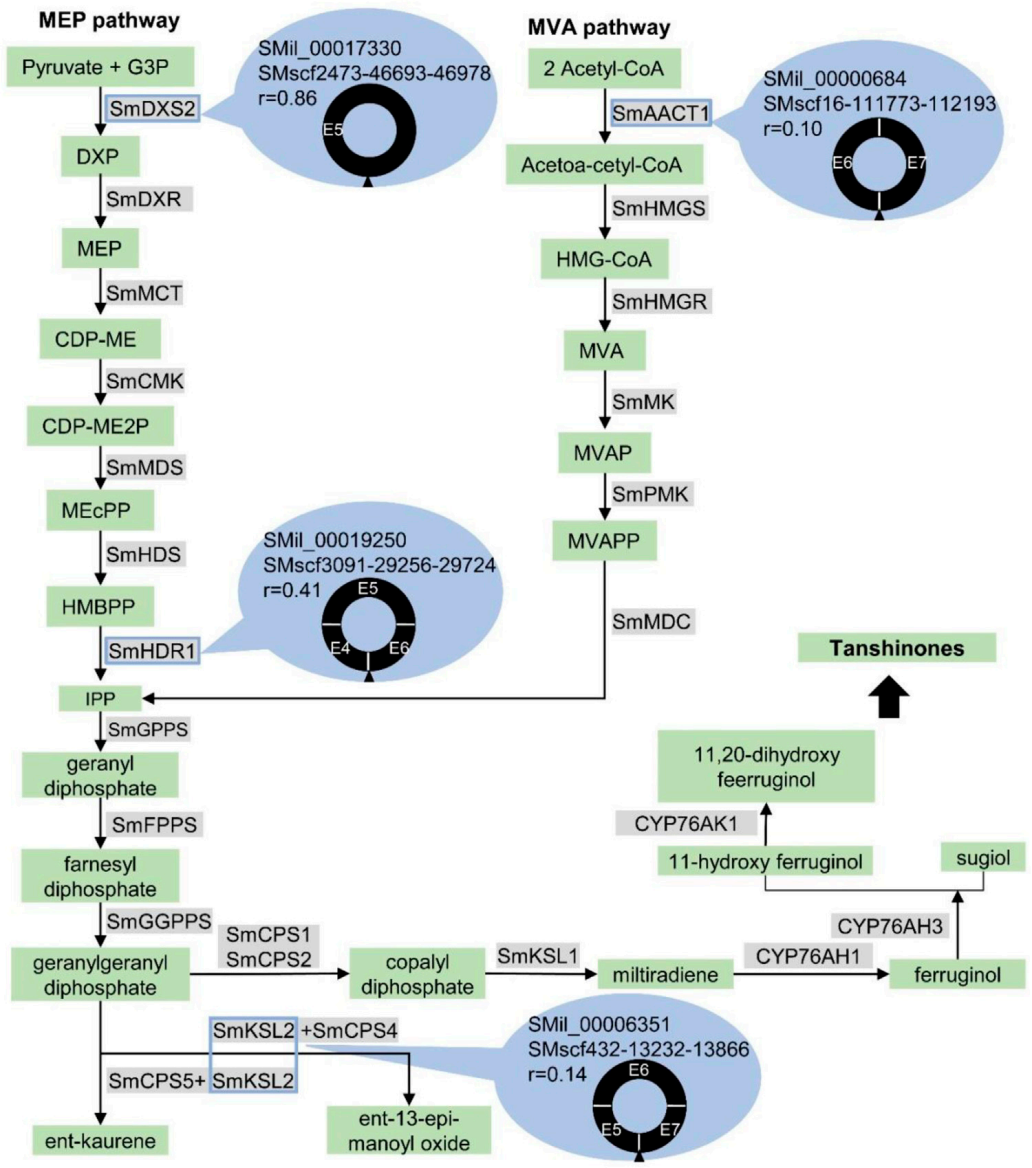
Potential involvement of circRNAs in the tanshinone biosynthesis in *S. miltiorrhiza*. The enzymes and chemical compounds were shaded in gray and green, respectively. Solid arrows indicated the reactions that are experimentally validated. Dashed arrows indicated the hypothetical reactions. The genes that produce circRNAs, SmAACT1, SmDXS2, SmHDR1, and SmKSL2, are marked by blue rectangles. The gene ID, circRNA ID, the expressed correlation between gene and circRNA, and the structure of circRNAs were shown in blue ovals. The exons are labeled with “E” followed by the exon number. The black triangle indicates the junction site.

The circRNAs can regulate the expression of parental genes (Hansen et al., 2013). Here, we found that the parental genes of six validated circRNAs were mapped to the biosynthesis of secondary metabolites. The first circRNA was the *S. miltiorrhiza* 1-deoxy-d-xylulose-5-phosphate synthase 2 (SmDXS2) gene. The encoding protein, DXS, was the first enzyme in the MEP pathway that catalysed pyruvate and glyceraldehyde 3-phosphate (G3P) to form 1-deoxy-D-xylulose 5-phosphate (Figure 8) (Zhou et al., 2016). The SMscf2473-46693-46978 circRNA originated from the circularisation of exon5 of the SmDXS2 gene, and it was experimentally validated (Figure 2B). The SmDXS2 gene and SMscf2473-46693-46978 had the highest and lowest expression level in the roots and stems, respectively (Figure 6A). The correlation coefficient of this circRNA and its parental gene was 0.86 (*p* = 0.003), indicating a strong correlation.

The second circRNA was the *S. miltiorrhiza* 4-hydroxy-3-methylbut-2-enyl diphosphate reductase 1 (SmHDR1) gene. HDR catalysed 4-hydroxy-3-methylbut-2-enyl diphosphate to form IPP, which is the precursor of all terpenoids (Figure 8). The SMscf3091-29256-29724 circRNA originated from the circularisation of exon4 and exon6 of the SmHDR1 gene, and it was experimentally validated (Figure 2C). The correlation coefficient of this circRNA and its parental gene was 0.41 (Figure 6B), indicating a moderate correlation.

The third circRNA was the *S. miltiorrhiza* acetyl-CoA C-acetyltransferase 1 (SmAACT1) gene. AACT was the first enzyme in the MVA pathway that catalysed two acetyl-CoA molecules to form acetoacetyl-CoA (Figure 8) (Chappell, 1995; Cui et al., 2010). The SMscf16-111773-112193 circRNA originated from the circularisation of exon6 and exon7 of the SmAACT1 gene, and it was experimentally validated (Figure 2D). The correlation coefficient of this circRNA and its parental gene was 0.10 (Figure 6C), indicating a weak correlation.

The fourth circRNA was the *S. miltiorrhiza* kaurene synthase-like 2 (SmKSL2) gene. Collectively, SmCPS5 and SmKSL2 can catalyse geranylgeranyl diphosphate (GGPP) to form ent-kaurene, which is the diterpene precursor to gibberellins (Figure 8) (Cui et al., 2015). By contrast, SmCPS4 and SmKSL2 can catalyse GGPP to form ent-13-epi-manoyl oxide (Cui et al., 2015). The SMscf432-13232-13866 circRNA might originate from the circularisation of exon5 and exon7 of the SmKSL2 gene, and it was experimentally validated (Figure 2E). The correlation coefficient of this circRNA and its parental gene was 0.14 (Figure 6D), indicating a weak correlation.

The fifth circRNA was SMscf7007-10563-10888 (Figure 2F). Its parental gene was homologous to *Arabidopsis thaliana* DWF4 (AT3G50660), which encoded a 22alpha-hydroxylase, and it was involved in the brassinosteroid biosynthetic pathway (Choe et al., 1998). The correlation coefficient of this circRNA and its parental gene was 0.09 (Figure 6E), indicating a weak correlation.

The last circRNA was SMscf1730-1749-2013 (Figure 2G). Its parental gene was homologous to *Arabidopsis thaliana* CYP88A3 (AT1G05160), which encoded an ent-kaurenoic acid hydroxylase in the gibberellin biosynthetic pathway (Helliwell et al., 2001). The correlation coefficient of this circRNA and its parental gene was 0.44 (Figure 6F), indicating a moderate correlation.

## Discussion

Many circRNAs have recently been studied in animals and plants for their potentially important functions in gene expression regulation (Zhao X. et al., 2019). However, reports on circRNAs in non-model plants are limited (Zhao W. et al., 2017; Zhang X. et al., 2019). Here, we reported the first genome-wide identification and characterisation of circRNAs in *S. miltiorrhiza*, an important medicinal plant. Using high-throughput DNA sequencing technology (Supplementary Table S1), we identified a total of 2,476 circRNAs from three types of plant tissues (Supplementary Table S2). In the validation experiments, 31 out of 55 circRNAs were validated successfully (Supplementary Table S3). GO term and KEGG pathway enrichment analyses showed 35 GO terms, and five KEGG pathways were significantly enriched (Supplementary Table S4). One hundred fifty-five circRNAs were significantly and differentially expressed amongst the three tissues (Supplementary Table S5). In addition, the expression profiles of 305 circRNAs correlated significantly with their parental gene (Supplementary Table S6). Amongst the genes involved in the biosynthetic pathways of active components, the expression profile of one of the genes significantly correlated with parental genes. Finally, we found 416 exonic circRNAs potentially correlated with 690 miRNAs (Supplementary Table S7). Moreover, we aimed to investigate the circRNA involved in the secondary metabolism, and the enrichment analysis results indicated that the circRNAs might be involved in the primary metabolism, particularly the carbon metabolism. One possibility is that the genes involved in the primary metabolism are expressed relatively at high levels, which result in the production, either specifically and non-specifically, of more circRNAs. This hypothesis will be an interesting topic to address in future studies.

The number of circRNAs identified in plants differed significantly (Lai et al., 2018; Zhao X. et al., 2019). Here, we identified a total of 2,476 circRNAs in *S. miltiorrhiza*, which were less than those in *O. sativa* (12,037) and *A. thaliana* (6,012) (Ye et al., 2015) and more than those in tomato (854) (Zuo et al., 2016) and peanut (347) (Zhang X. et al., 2019). The identification of circRNAs depends on the software used. Thus, focussing on the circRNAs that are found by using different software tools is important. We used different software tools to predict circRNA. The results can be found in Supplementary Table S9 and Supplementary Figure S4.

CircRNAs identified in *S. miltiorrhiza* also showed significant variations across three biological replicates. All three replicates shared only 16.56–22.47% of all identified circRNAs. The low replication rate may be due to the low expression level of circRNA, insufficient sequencing depth and high degree of genetic diversity amongst the replicated tissues. This phenomenon was also found in other studies. The number of shared circRNAs in biological replicates was 17.34% in *O. sativa* (Lu et al., 2015) and 20–26% in *G. lucidum* (Shao et al., 2019). In addition, 56% of circRNAs were validated successfully using PCR and Sanger sequencing, which indicated that circRNAs were present in *S. miltiorrhiza*.

The expression of circRNAs was considered as tissue-specific (Ye et al., 2015). In this study, the number of circRNAs shared amongst three tissues (root, stem and leave) was, on average, 12.01%, which was less than those in kiwifruit (17.97%) and more than those in soybean (2.7%). In addition, the origin of circRNAs was different in plants (Chu et al., 2018). In this study, most circRNAs (84.65%) were generated from exon, similar to those in *A. thaliana* (85.7%) (Ye et al., 2015) and *Gossypium raimondii* (84.24%) (Zhao T. et al., 2017). By contrast, the number of intergenic circRNAs was more than the number of exonic circRNAs in kiwifruit (51–67%) (Wang et al., 2017) and *Triticum aestivum* (60.2%) (Wang et al., 2016).

The reported circRNAs can regulate the expression of their parental genes. Here, the expression profiles of 286 circRNAs correlated positively with their parental genes. The circRNAs may positively regulate the expression of parental genes by acting as miRNA sponges (Kristensen et al., 2019). MiRNAs can inhibit the expression of target genes by binding and cleaving the mRNA in plants (Yu et al., 2017). CircRNAs can bind specific miRNAs, preventing them from regulating target genes (Memczak et al., 2013). Here, we found 416 exonic circRNAs that might interact with 690 miRNAs. However, the identification of miRNA targeting was based on computational predictions. The reliability of these predictions needs further investigation. By contrast, the expression profiles of 19 circRNAs correlate negatively with their parental genes. The circRNAs may negatively regulate the expression of parental genes by regulating the splicing of mRNA. For example, circRNAs derived from exon 6 of the SEPALLATA3 gene can decrease the abundance of mRNA, which contains exon 6, but can increase the abundance of the mRNA, which lacks exon 6, in *Arabidopsis* (Conn et al., 2017).

Six out of 31 validated circRNAs are originated from parental genes involved in the biosynthesis of secondary metabolites. The SmDXS2 gene can encode 1-deoxy-d-xylulose-5-phosphate synthase, which is the first enzyme in the MEP pathway. The SmHDR1 gene can encode 4-hydroxy-3-methylbut-2-enyl diphosphate reductase, which produces IPP (the precursor of all terpenoids). The SmAACT1 gene can encode acetyl-CoA C-acetyltransferase (AACT), which is the first enzyme in the MVA pathway. The SmKSL2 gene can encode kaurene synthase-like cyclases, which produce ent-kaurene (the precursor of gibberellins). The SMil_00026090 gene can encode 22alpha-hydroxylase, which is involved in the brassinosteroid biosynthetic pathway. The SMil_00014508 gene can encode ent-kaurenoic acid hydroxylase, which is involved in the gibberellin biosynthetic pathway. Amongst them, the SmDXS2 gene showed a significantly positive correlation (*r* = 0.86, *p* = 0.003) with its circRNA SMscf2473-46693-46978. Tanshinone is a diterpene compound and the main active compound of *S. miltiorrhiza* (Ma et al., 2015). The SmDXS2 gene and its circRNA had a significantly higher expression level in the root than in the leaf and stem, which is consistent with the accumulation of tanshinones in *S. miltiorrhiza*. Consequently, SMscf2473-46693-46978 might regulate tanshinone biosynthesis by regulating SmDXS2 expression. These results indicate the potential involvement of circRNAs in the biosynthesis of secondary metabolites in *S. miltiorrhiza*. Future studies are needed to explore the potential functions of circRNAs.

## Data Availability

The datasets presented in this study can be found in online repositories. The names of the repository/repositories and accession number(s) can be found in the article/Supplementary Material.

## Abbreviations

AACT1, acetyl-CoA C-acetyltransferase 1; CircRNA, circular RNA; DXS2, 1-deoxy-d-xylulose-5-phosphate synthase 2; GO, gene ontology; HDR1, 4-hydroxy-3-methylbut-2-enyl diphosphate reductase 1; IPP, isopentenyl diphosphate; KEGG, kyoto encyclopedia of genes and genomes;KSL2, kaurene synthase-like 2; MEP, methylerythritol phosphate; MiRNA, micro RNA; MVA, mevalonate; WGCNA, co-expression network analysis
