# Supplementary material for: Genome-Wide Identification of Circular RNAs Potentially Involved in the Biosynthesis of Secondary Metabolites in Salvia miltiorrhiza
Source: Front Genet. 2021 Nov 5;12:645115. doi: 10.3389/fgene.2021.645115 (PMC8602197; doi:10.3389/fgene.2021.645115)
Supplement: Supplementary file 1 [file DataSheet1.ZIP › Supplementary_Material/Supplementary_Material_1.docx]

Supplementary Material

# Supplementary Figures


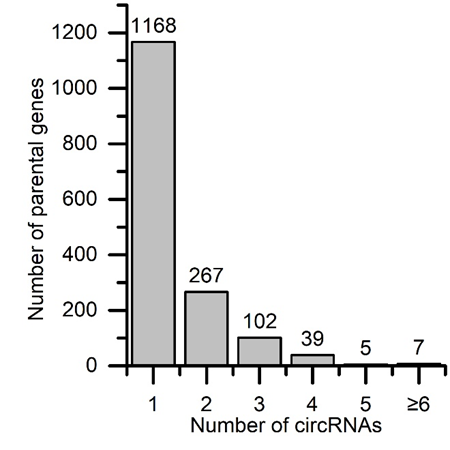


**Supplementary Figure 1.** The number of parental genes producing circRNAs of different amounts. The x axis shows the number of circRNAs produced from one parental gene.


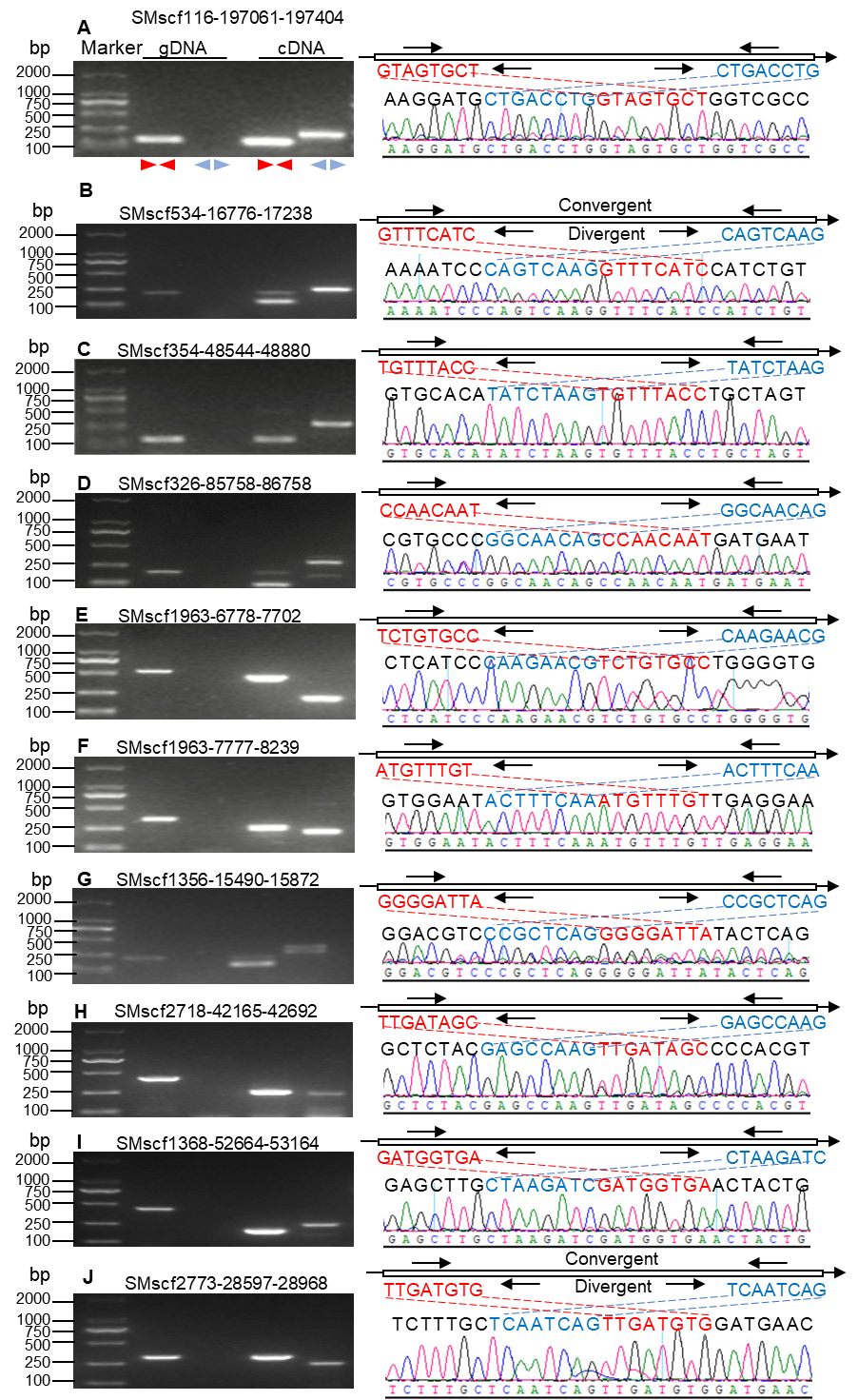


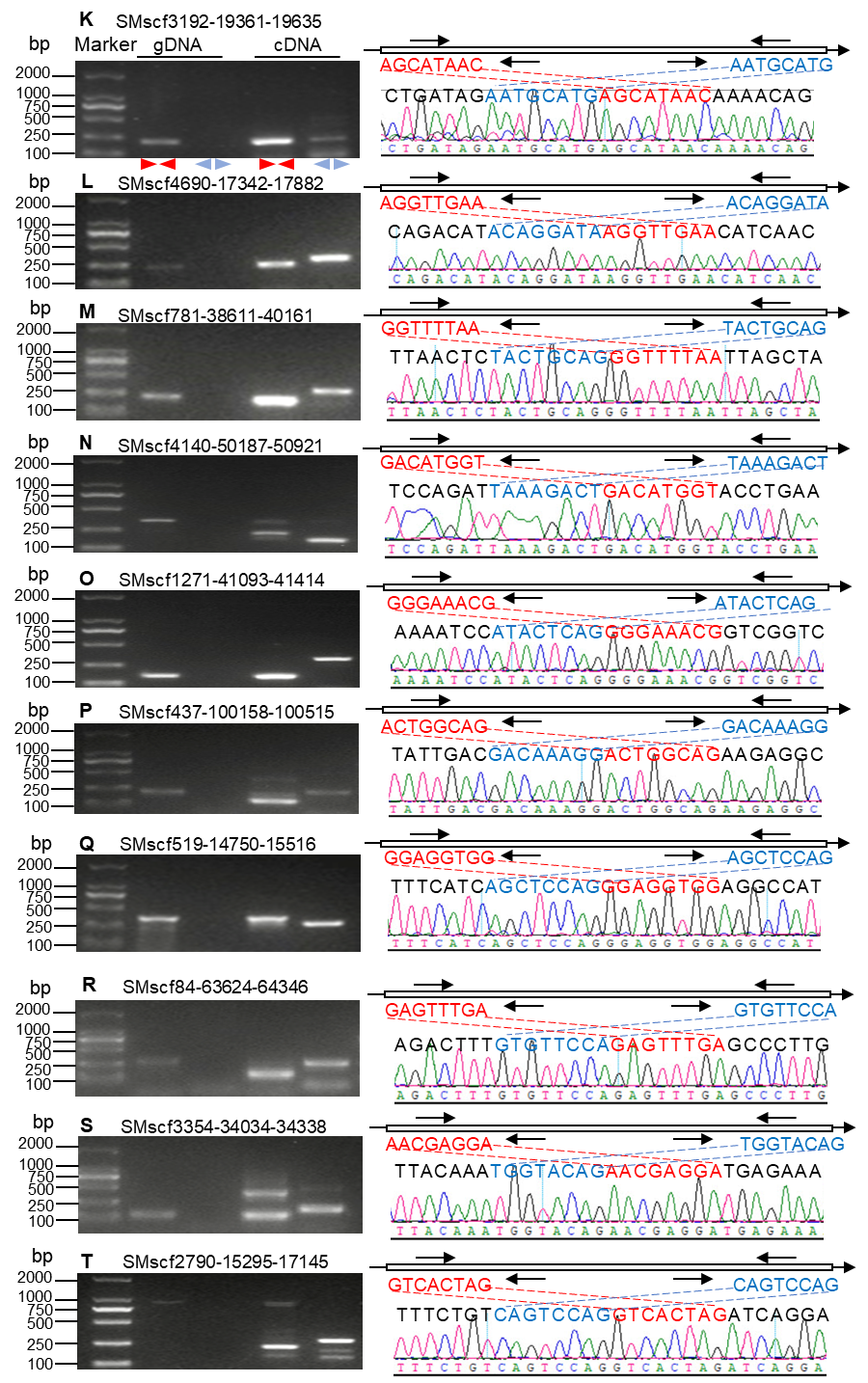


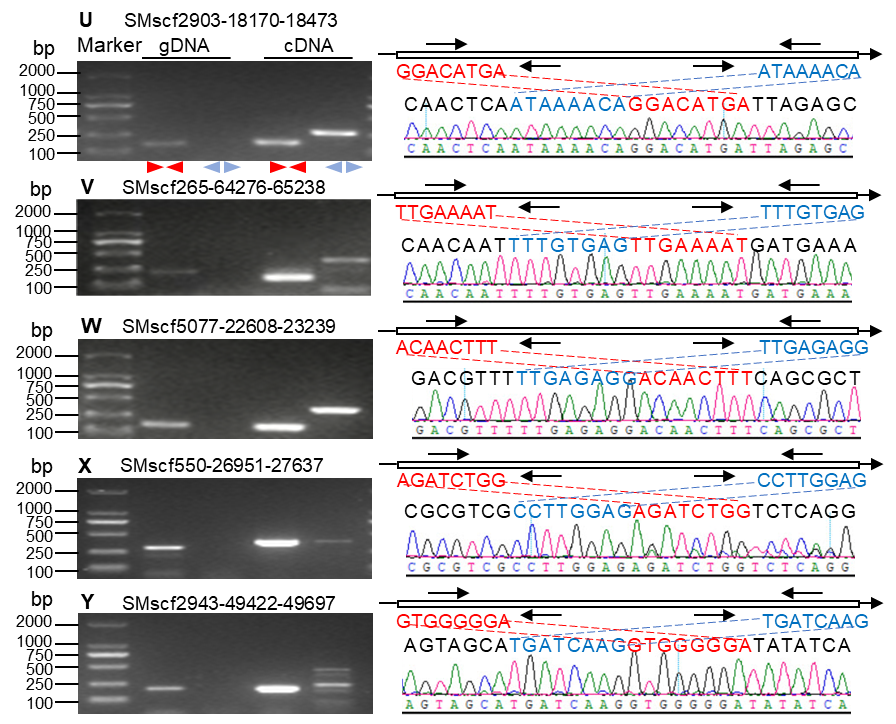


**Supplementary Figure 2.**  The left part in each panel is the electrophoretic gel picture of the PCR results. The type of template for PCR is shown on the top of each gel picture. The type of primer is shown at the bottom of each gel. gDNA: genomic DNA; cDNA: complementary DNA; "”: convergent primer pair; “": divergent primer pair. The right part in each panel compares the sequences around the junction sites with those obtained from Sanger sequencing. The upper right part in each panel shows the schematic representation of the sequences around the junction sites of the circRNAs. The thin line represents the genome sequence. The start and end of the unfilled rectangle indicate the expected junction site of the corresponding circRNA. The red sequence locates downstream of the junction site, while the blue sequence locates upstream of the junction site. The convergent and divergent primer pairs are shown above and below the genomic sequence, respectively. The mapping of the sequences around the expected junction sites and the sequences obtained from Sanger sequencing are connected with dashed lines.


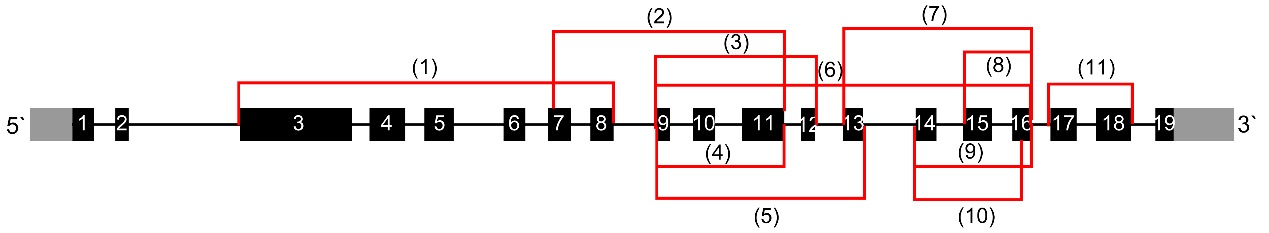


**Supplementary Figure 3.** The structure of 11 circRNAs originated from parental gene SMil_00000685. The gray and black rectangles represent UTR and exons, respectively. The black lines between exons represent introns. The red lines linked the back-splice sites. The numbers displayed above or below the red lines represent the circRNAs, (1) SMscf16-116930-118041, (2) SMscf16-117787-118624, (3) SMscf16-118107-118890, (4) SMscf16-118107-118624, (5) SMscf16-118107-119061, (6) SMscf16-118107-119780, (7) SMscf16-118967-119780, (8) SMscf16-119440-119780, (9) SMscf16-119152-119780, (10) SMscf16-119152-119776, (11) SMscf16-119851-120805.


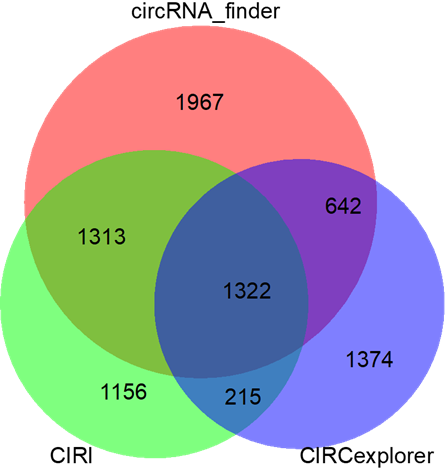


**Supplementary Figure 4.** Comparison of circRNAs identified by three software.

# Supplementary Table 1-9 is provided in the excel sheet.

Table S1 Summary of the RNA-seq data.

Table S2 The identified circRNAs by both circRNA_finder and CIRI softwares with at least two junction reads in *S. miltiorrhiza*.

Table S3 Primers used for the validation of circRNAs.

Table S4 GO terms for the parental genes of circRNAs.

Table S5 Differentially expressed circRNAs across the three tissues in *S. miltiorrhiza*.

Table S6 Correlation for expression profiles between circRNAs and their parent genes in *S. miltiorrhiza*.

Table S7 The information of circRNAs with miRNA-binding sites and miRNAs in *S. miltiorrhiza*.

Table S8 Significantly enriched KEGG pathway for modules had the significant correlation with circRNAs.

TableS9 The circRNAs identified by CIRCexplorer software with at least two junction reads in *S. miltiorrhiza.*
