# Supplementary material for: Genome-Wide Identification of Circular RNAs Potentially Involved in the Biosynthesis of Secondary Metabolites in Salvia miltiorrhiza
Source: Front Genet. 2021 Nov 5;12:645115. doi: 10.3389/fgene.2021.645115 (PMC8602197; doi:10.3389/fgene.2021.645115)
Supplement: Supplementary file 1 [file DataSheet1.ZIP › Supplementary_Material/Supplementary_Material_3.docx]

>SMscf1963-6778-7702

TCTGTGCCTGGGGTGCCTGAGATCAAAGAAGGCTACAATCCAGCTACTTGGATGTTGGATGTCACCACTCCTTCCATGGAAGCTCAACTCAACGTCGACTTTGCTGAAATTTACTCCAAATCTGAGCTGTACCGGAGGAATGAACAACTCATCGAAGAGCTGAGTAGTCCAGCACCGGGTTCAGAAGATCTGCATTTCCCGACCCAATACGCGCAGTCCTTCTTTGTTCAGTGCAGAGCTTGCTTCTGGAAGCAGAACTTGTCTTATTGGAGGAACCCTCAATATAATGCCATCAGATTCTTCATGACAGCACTCATCGGTGTCATATTCGGGGTCGAAGCAGCAAGACCTGATGAACCTGCTGGGAGCCATGTACTCTGCAGTGATGTTTCTTGGTGGCACGAATACGTCTGCTGTCCAGTCCGTGGTGGCCGTGGAGAGGACTGTGTTCTACCGCGAGAAGGCAGCAGGGATGTACTCGCCGTTGCCCTATGCATTCGCACAGGTGGCAATCGAGACACTCTATGTGTCGGTCCAGACCTTAATATACAGCCTCATCCTGTATCCATGATTGGCTTCCACTGGAGCGCGGACAAGTTCTTCTGGTTCTATTTCTTCGTCTTCATGTGCTTCGTCTACTTCACCTCGTACGGGATGATGCTCGTTGCCCTCACCCCCAACTACATGATTGCTGCCATCGTCATGTCCTTCTTCCTCAGCTTCTGGAATCTCTTCTCCGGCTACCTCATCCCAAGAACG

>SMscf1963-7777-8239

ATGTTTGTTGAGGAAGTGATGGATTTGATTGAGTTGAATCCTCTGAGGAATGCCTTGGTTGGGCTTCCCGGAGTGGACGGTCTGTCGACTGAGCAGAGAAAGAGACTGACCATAGCAGTGGAGCTGGTTGCCAATCCATCCATAATCTTCATGGACGAGCCTACATCAGGGCTGGATGCACGAGCTGCTGCTATTGTGATGCGAACTGTGCGCAACACGGTGGATACTGGACGGACCGTTGTCTGCACCATCCACCAACCCAGCATAGATATCTTTGAAGCCTTTGATGAGCTATTGTTGATGAAGAGAGGAGGGCAGGTGATTTATGGAGGGCCTCTTGGCCATCACTCTCGCTTGTTGGTGGAATACTTTCAA

>SMscf3091-29256-29724

GTTTGGAATACTGTTGAGAAGCACAAGAAAGGAGAGTATACTTCTATTATCCATGGTAAATATTCTCATGAAGAGACTGTGGCCACTGCTTCTTTTGCGGGGAAATTTGTCATTGTAAAGAACATGACAGAGGCAACATATGTATGTGATTACATCTTGGGTGGTGGACTTGATGGATCCAGCTCTACTAAAGAAGCATTTCTTGAGAAATTTAAATTTGCTATATCCAAGGGATTTGACCCGGACAAGGATTTTGAGAAAGTTGGTATTGCGAATCAAACTACTATGTTAAAAGGAGAAACAGAGGATATTG

>SMscf2718-42165-42692

TTGATAGCCCCACGTCCGTGTATACCTCAAAGGGCGCGGATAACCAAGCAGTTGGCGCTAGCAGTGGCTCGTCACACGACCAATCAGACGAAGAGGACATGGAGATAGAAGCAGGCTCTTATGATCCCACCACCAATCCTATTGATGTCAAGCGCATCAAAAGGATGGTTTCCAACCGGGAGTCTGCACGACGTTCTAGACGAAGGAAGCAAGCACACTTAGCAGATCTTGAACAACAGGTAGAACAATTACGAGGAGAGAATGCAACTCTGTTCAAGCAACTCGCTGATGCTACTCAGCAATTCAAAGATTCGACAACAAACAATCGCGTGCTTAGATCAGACGTGGAAGCTCTACGAGCCAAG

>SMscf1368-52664-53164

ATGGTGAACTACTGGAAATCCAAGCTTCTTCCCAAGATCAAGAAGGTTTTCGAGAACCCCAAAAAGGCAGCCGGTCTCGAAGCTTGCAAGACCTTCGATGAATCTAAGGAGCAATATTCGAAAGAGTGTGAAGAGAAAAAGAGCGATCTTGAGCCCAAAGTTACCGAAATCTATCAAGCTTCATCTACTGAGATTAAGGCTTTAATCAAGGAACCAACGGATTCAGGTGTGAAGAAGCACTCGGCAGCAGTCCAGAAATTCCTCGACGAGCTTGCTAAGATCG

>SMscf2773-28597-28968

TTGATGTGGATGAACACAACCATGGCGGCAATCCGATGTACACGAGAGAGTTTCAGACAATGTTGGAGGGTTTGGAGGAGGAAGAATCTGGGCAGATTTCGGAGAAGAAAAGGAGACTGAGCGTGGATCAAGTAAAGGCTTTGGAGAAGAATTTTGAGGTGGAGAACAAGCTTGAGCCAGAGAGGAAAGTCAAGCTGGCGCAGGAGCTAGGCTTGCAGCCGCGGCAGGTGGCCGTGTGGTTTCAGAACCGCCGTGCGCGGTGGAAGACCAAGCAGTTGGAGAGAGATTACGGCCTCCTCAAAGCCAACCACGAAGCCCTCAAGCATAAATACGAGTCCCTCCACAGAGAGAATCAGTCTTTGCTCAATCAG

>SMscf16-111773-112193

GTGCTTTGAAGAGAGCAAATATCGATCCATCACTTGTACAAGAAGTTTTCTTAGGGAATGTACTCAGCGCAAACTTAGGACAGGCTCCTGCCCGTCAGGCAGCGTTGGGTGCCGGGATCCCGAATTCAGTAGTCTGTACAACCATCAACAAAGTCTGTGCCTCTGGAATGAAAGCAACTATGCTAGCAGCGCAAAGTATCCAGTTGGGTCTCAATGATGTTGTAGTGGCCGGTGGCATGGAGAGCATGTCTAATGTCCCAAAATACATCGCAGAGGCGAG

>SMscf2473-46693-46978

ATTGGGAAGGGAAGGATTTTAAAAGAGGGGAGTAGAGTTGCCATTCTAGGGTTCGGAACTATAGTGCAGAACTGTTTGGCGGCGGCGCAGCTTCTTCAGGAACACGGCATCTCCGTGACCGTAGCTGATGCGAGATTCTGCAAGCCTCTGGATGGAGATCTGATCAAGAAGCTGGTGCAGGAGCATGAAGTTCTCATCACTGTTGAAGAGGGATCTATTGGTGGATTCAGCGCCCATATTTCTCATTTCTTATCCCTCAACGGACTGCTCGACGGGAACCTTAAG

>SMscf3192-19361-19635

AGCATAACGAAACAGATAGGGCCGCAAGCGCATGAAGTCGCAGCCAAGGTGGATGAATACGCAAGGGGGATGCTGAGCGCATCTGGCTCAACCTTCTTTGAGGAGCTCGGGTTGTACTACATTGGGCCGGTGGATGGGCACAACATCGATGATTTGGTCACTATTTTTCAGAAGGTTAAGTCGATGCCTGCACCGGGGCCGGTCCTCATTCACATTGTGACGGAGAAGGGAAAAGGATATCCACCAGCAGAAGCAGCAGCTGATAGAATGCATG

>SMscf7007-10563-10888

GTACGGGAAGATATTCAAGTCCCATTTGTTCGGGGAGGCGACGGTGGTGTCAGCAGACGCAGGGCTGAACAGATTGATTTTGCAAAACGAAGGGAAGCTGTTCGAGTGCAGCTACCCAAGGAGCATCGGCGGGATCCTGGGGAAATGGTCGATGCTAGTGTTGGTGGGTGATATGCACAGGGACATGAGAAGCATCTCCCTCAACTTCCTCAGCACCTCCAGGCTCAAGACCCATCTCCTCCACCAGGTCCAGACACACACCTCCCTCGTCCTCTCCTCCTGGGCCCACGGCTCCGTGCTCTGCGCCCAGGACCAGGCCAAGAAG

>SMscf4690-17342-17882

GTTGAACATCAACGGAATGCATTAGAAATGAAACTTGGTGTATTGCTTGAGAGAGCTAGTTCAATTCCTGAACAGATATTGGTTCATGTCATAGAAAATGCTCAAAGTGCAAGGCCCAATGGAGCTGTGGACTCTGAAACAATTTCTTTTTATCTAGCTTATACCATCCTCGGAGCTACAATATTTGGAGATGTGTTTTTGGCTTGGCCTAAGGCTACTTTCTTTAAGGAGCTGCTAGTGAAGATAGCTAAAGATGCATGCTTTTGGGCATCTTATGGTGTCACTCCTTTCTGGAAAAGGGGTTTTTGGAGGTACCGGGATTCTTGTGCTAAGTTGAAGTCCTTAATGCAGGAGCTTATAACAGAATGCAGACAAAACTGCAAGTCACGATTTTTATGGGAGTTTGATGCCCATGATCACACGGAACATGAGCCATGTGGGGAAATAATAAGTCTAATGGTCCATGGGTCTCTGACTATGGCTGCTCTTATTTCTAATATCTTGACAAGGCTTGTGACACATGCAGACATACAGGATAAG

>SMscf781-38611-40161

GGTTTTTATTAGCTACTGCCAGTTTAGGAGATGTACGAGAATTGGGGCGGTTTTTTCCGAGAAATTTGAAGAGTTGTAGTTGACTAGTTGCTAGTTGTTTGGCATTTTCTGAGTAAAGTTCATTTGGGTTTTAGAGATTATACATATTGGAGGGTGTGGATGATGGATAAGGATAAGTCTCACCATGGGAACATACTTCCGCCGTCGGGGAGGTACGCTGCTTTCTCGCCTCCGGGGAGTAGTTACAATGCGAAGTCGGAGCAAGCTGGTTTGTCGAGCTTGCCTCCGTTGGGGCCGGGCAGTTCATCGGAACAGGGTCATTTTGGTCATGGCATGCCGTCGGATTCTAGACAGTTTAGTCATGATATAAGCCGCATGCCCGACAATCCACCGAAGAATATGGGTCACCGGCGTGCTCACTCTGAGATTCTCACCCTTCCGGATGATATTAGCTTTGATAGTGATTTGGGTGTTGTTGGCGGTTTGGATGGGCCATCCTTTTCCGATGATACGGAGGATGACTTGCTCTCGATGTATCTCGACATGGATAAGTTCAATTCGACGTCTGCAACTTCATCATTCCAAGTTGGTGAGTCGTCCAATGCAATGGCAGCAGAGCAAGGTCTTTCGTCTGGTCCTTTGGGAGCAGCTAATCAATCTTTAAGTGATAAACCAAGGATTAGGCATCAGCATAGCCAGTCCATGGATGGGTCGACCACTATCAAACCAGAGATGCTCATGTCTGGGCCAGAAGATCCAGCTTCTGCTGATGCTAAGAAGGCCATGTCTGCTGCTAAGCTCGCTGAGCTTGCTCTTATCGATCCAAAGCGTGCTAAGAGGATTTGGGCAAATAGGCAGTCAGCAGCAAGATCAAAGGAGCGTAAGATGAGGTATATAGCTGAACTTGAAAGGAAAGTACAGACTCTGCAAACAGAAGCAACTTCGTTGTCTGCACAGTTAACTCTACTGCAG

>SMscf1730-1749-2013

GCGCGTAAACGGCTGGTGGCTGTTCTCCAATCTATAGTAACTGCACGAAGGGAGTGGAGGACGAAGAACCCTGTAGGGTCGAAGAAAGACATGATGGACGCGCTGATGGATGCTCAAGATGAAAAGGGTAGAAGCTTGAAAGACGAGGAGATCATTGATGTTTTAGTTATGTATCTCAACGCGGGCCATGAATCGTCTGGACACATCACTATGTGGGCGACCCTCTTCCTGCAGAACCATCCCGATGTCCTCCAACGAGCTAAG

>SMscf326-85758-86758

CCAACAATGATGAATTCCACATCCACACAATTTGTTCCCTCAAGACGGATGGGCATATATGAGCCGATCCATCAGATGGGCATGTGGGGAGATTTCAAAGGTAACAATTGTCTGGATGCATCTCCACCGATGATTCTGGAGGTTGACCCTAAACTAGATAATCAGTCAGAGGATACTTCACATGGAACTGTTGGACCTTCAAATAAATACGACCAAGAAGCAAGTAAACCACCTGATAAGGTTTTAAGGCGTCTAGCACAGAACCGTGAGGCTGCTCGTAAAAGTCGTCTAAGGAAAAAGGCCTATGTTCAGCAATTAGAAAGCAGTAAACTAAGGCTTATTCAGTTAGAGCAAGAGCTCGGCCGTGCTCGGCAACAG

>SMscf4140-50187-50921

GACATGGTACCTGAAATAACTGCTTGTGTTGAAACCATGCTTGAGAAATGGAGGAATTACCATGGCAAAGAAATCGAGGTTTGCAGCGAATTCAAGCATCTTAGCTCCGAAGTAATTTCAAGAACAGCTTTTGGAAGTAACTGTTTGGAAGGGAGAAAGATATTTGATATGTTGTCAAAGCTTATTGTATTGATTTCCAGAAATATGTTAAAGATCAGACCTTTTGGCCTTGGAAAATTTATAAGGACTCAGGATGACAAGGAAGCAGACAAAATCGAGCAGTTGCTTCATGATTCTATCATGGAAATAGTAAACAAGAGACAAGATGAAGTTCAAACTGGGAAAGCAGACAGTTTTGGGAATGATTTTCTAGGATCACTCCTAAAATCTCACCACGACACGGATCCGAAGAGCCATATATCAGCAGCTGAGATCATTGATGAATGTAAAACCTTTTATTTTGGTGGACAAGAAACAACTTTTAGTTTACTATCATGGACCACATTTCTCCTTGCAATCCACACAGAGTGGCAAGACAGAGCCAGAGAAGAGGTGTTGCAGCTATTCGGTCAGCAAAACCCCAATTCAGATGGCCTTTCCAGATTAAAGACT

>SMscf1271-41093-41414

GGGAAACGGTCGGTCTCTGATCACATGCACGAGGCTGCAAGTTACATAAAGGATATGGAGAAGAAGATAGAGGAATTGCAGTTGCGGAGAGATGAGCTGAAAAATGCATCGGCTTCAAATACTACTTTTGATCTGCCAAATTTTGTAACGGTGAATTGTTGTTGTGATGGGCTTGAGATTTTGATCAACTGTGGTGTCAAAGAGGGTTATGGATTTTCCCTATCAAGAGTGATTGTGGAATTGACTCACAACGGACTCGATGTAGTCACCTGCATTTCAAACAAAGTGAATGGCAGATTCTTGTACAAAATCCATACTCAG

>SMscf437-100158-100515

ACTGGCAGAAGAGGCTACAAATGGGAACCCGGGAGGGCCCGATGCTCGGACGCTCAAGATGATATCCAAGGCCGCTTTCGAGGTCGATGATTATTGCAGAATTGTCGACATCCTTCACAAAAGATTTGAAAGATTTGAGAGGAGAAATTGGAGGGCATCGTACAAGGCTCTGATGGTGCTGGAGCACTTGTTGACTCATGGCCCCGAGAGAGTTGCAGAGGAGTTTCAAGGCGACAGAGATGTGATCGCACAAATGGACAACTTCCATCATATTGACGACAAAGG

>SMscf354-48544-48880

TGTTTACCTGCTAGTCCAATGGGAAGCGGTCATCATCGGTCCACCAGTGAGAGTAGTTCATTTGAAAGCGCCGACTTTGATCATGGAGCAATTGAAGAGTACACATCAGAGAGATTTAACTCATTACAGATAGACCGTCCTGTGAGAAACTCACAACAGCAAAGAACTGGTGTAAAAACCGAGAAGCCAATCGATGTACTGCGGAAAGTTTGCGGTAATGACAAATGTGCTGATTGCGGTGCTTCTGAACCTGATTGGGCATCCTTGAATCTGGGCGTTCTTGTATGCATAGAATGCTCTGGCGTTCACCGTAATCTTGGTGTGCACATATCTAAG

>SMscf519-14750-15516

GGAGGTGGAGGCCATTTCCAAACATCCTCACGCTTCCAGTAGAAGGGCTGTTCGAATTAGAGAAACTGTTTCATTAATTAGAGTGCACGCTGTGATGAGTGAGCTTGTTGGCAGCTCTTGCGGCGTTCTTAGTTCTTCTTCAATGACCTGAAATGCGAGGGTGAGGATACTATTCTGAGTCGAGGAACCATCCGGTAGAACGACGAGCCCCGAAGGCAAGATTGCCACGGAATCTGCACTCCCTCCTTGCAGAATGCGATGCATGGTCGGGACATCTATGGGAGCGTAGACTAAGTAGCATGCCACAGAGTCGCTGTAGCTTTCTTGCAGATACATGTGGCCAAAGGAAATAAGATTAGCAGGTATGTGGAACTACCCCTACTGAAACTACTGAAACAGAGTCTGTGGAGTTGCGAGCTGAAGAAACGCGTACAACATCTTGAACTTCCAGACCATGGGATAGAATATCCCACTGCAGCGTATCTGTAAGGTAGCCAAACTGAAGTTGCAATCGACACAACTACTCGACAAGGGATTTGCAGATCATCTAGGTTGAAGCTGGTCTTGATCAGTATTTCATCAGCTCCAG

>SMscf84-63624-64346

GAGTTTGAGCCCTTGATACAGAAGCCTTACTCAATAGTTTATTTCCACTCAGCTGCATCCCTGCAGATACAACCAGATTTGGGGTGGATGAAAAGATTAGAGCAAATACTTGGTCGGAAACACCAACGCAATCTCCATGCGATATACGTCCTCCATCCAACATTTGGTCTGAAGACTGCAATATTCGGATTGCAGATGCTTGTAGATAATGTGGTCTGGAAGAAGGTGGTCTACGTTGATCGTCTTCTGCAGCTATTCAGATACGTCCCCCGTGAACAACTGACTATTCCAGACTTTGTGTTCCA

>SMscf3354-34034-34338

AACGAGGATGAGAAAATGAGACGAGAGTGGCAGAGGAAAGCGGCATTAGTCCTGCCTACAAGCGGAAACACAAGGTTGCTTTCTGCTCTATCCGACTCCCTTGCCAACAGCATCCCAAGTCTGGCTCGAGCTTGCCTCATTACAATCGGCTGGATCAGCCATTCGCTTGCTGATACAGATCTTCGCCTTGCAGCATGCTCCATTATCGCCCCGCGCTTGATGGAATGCCTCGTAGACAACACCAACAACCTTGAGGAAAAGATTCTGGCCTCATTTTCATTACATAGTCTTACAAATGGTACAG

>SMscf534-16776-17238

GTTTCATCCATCTGTGAATCGACTGTACTGCCCCTCAGAGGAGGTGGCAAAGAGGGCTTTGCTAGATGGTCTTCAAGAGTCTCAAATTCGAGTATTTGGCTTGCCCATTCGACCATCCTTTGTTCAAGCAGTTCTGTCCAAGGATGATTTGAGGATAGAACTCGAGATGGACCTGCAGTTGCCAGCTGTTCTGCTGATGGGGGGCGGAGAAGGCATGGGGCCGGTTAAGGAAACAGCAAAGGCTCTTTCAGAATCTCTGTTCGACACAGAACTTGAGAAGCCGATAGGCCAACTGATCATCATCTGTGGTCGTAATGAGATGCTAGCTTCCACATTGCAGTCACTTGAGTGGAAAATCCCAGTCAAG

>SMscf2790-15295-17145

GTCACTAGATCAGGATCAGTGCCTCCAAGCACAAGAGATGCTTTATACCAGGGATTGCCGCCAACTATAAAGTGTGCTTTGCGTTCCCGACTGCAATCTTTTCAACTCAAGGAAGAGTTTACTGTCCCACAGATCAAAGAAGAAATGGAGAAAACTCTCCAATGGCTGGTGCCTATGGCCACCAATACCACAAAGGCTCACCACGGCTTTGGTTGGGTTGGCGAATGGGCAAATACTGGATCTGAGATGAACCACAAACTTGCTGGCCAGACGGATTTGCTCCGTATCGAGACACTTTATCACGCGGACAAGGAGAAAACGGAAGCTTACATTCTTGATTTGGTACTGTGGCTGCATCATCTCATCAGCCAATCAAGGGCCGCCAATGGTGGGCTCAGATCCCCCGTCAAATCCCCGATCAGGTCCCCAAACCAGAAGACCCTCCAGCTGTCAAGCCGGAAACCGAGCTCGCCGTCGTCGTCGACTTTGTCGGTCGAGGACCAGGAGATGCTCCAAGATGTGAGCAAGCGGAAACTGACTCCCGGGATAAGCAAGAGCCAAGAATTCAACACGTCGAGAACCCGATTGTCGAAGCACCACAGGCTAACCAAGAGCAGCAGCCATTCTCCGACACACGAAACCACCAAGAGGGACCCTTTCCCCATCCGAAGGCCATCCTCTGTTCCGATCATCAACTTCGACATCGACAGGATCAAAGCGCTGGACATGATCGATAGAGCGCCTGCTCGTCCGTGGTTTGCAGTTGGCATCGTTTCTGCTGTAAATGAGCTCTGCGCCCTCCCATCCGCGCTCTTGCTGGGATCAGAGAGCCGCGGCCGTTGCAGCACCGTGTGGTACATGGAGAATTTGTGTCTTAGGTATACTGTTTTTTCCCGTGAGATCGAGAAAACCGGCAAGATCCAGCGGTGTTCGACCGGCGTTTCTGTCAGTCCAG

>SMscf2903-18170-18473

GGACATGATTAGAGCCATACGTGCATGCAAAACTGCAGCCGAGGAACGAGCTGTTGTAAGAAAAGAATGTGCTGCAATTCGAGCTGCAATTAGTGAAAATGACCAAGATTATAGCCATCGCAATTTGGCGAAGCTCATGTTTATTCACATGCTTGGATACCCAACACATTTTGGTCAAATGGAGTGCCTGAAGTTAATTGCATCACCGGGATTCCCAGAGAAGAGAATAGGATACCTTGGCCTTATGTTGCTTCTTGATGAAAGACAGGAAGTTCTGATGCTTGTCACCAACTCAATAAAACA

>SMscf1356-15490-15872

GGGGGTTATACTCAGGGGGAAGTGCTCAGATGGTTGAGAATTCACTTGGCATTCATGGAGATGAAATTATGTATGTTGGAGATCATATATATACAGATGTTAGTCAATCGAAAGTTCATCTTCGGTGGCGTACAGCACTAATTTGTCGAGAACTGGAAGACGAGTATAGTGCTTTGATTCGTAGTCGAGGTCATCGTGCCACACTGATAGAGCTCATAAATCAGAAGGAGGTTGTAGGAGACCTCTTCAACCAACTCCGCCTTGCTCTGCAAAGACGAAGTAAAGGACGTCCCGCTCAG

>SMscf265-64276-65238

TTGAAAATGATGAAACAATTGGAAAAGGCTCTGGATGGGAGCGTTTTGAGTTCGACAAGGATGCCCCTCTCGATGATGAGGAGGACATAGAAGCTGCTGATGACGATGCTGTGTTGGTCAAGAACATGGGACAAAGCTTTCGTTTCTCTGCAGTGGCGGCGAGGAAAGAGGAAGAGATGAAGGCCCTTCACGATGAAGCGATCTTTGGAGTCTCTTCACACCCACCGCCTGCAGAGACAGACAACGAGGCACAAGGTGCGAACCATGGAGATGAAAACGTTGAAACTGCTCCTGCAATGAGTCTTCTCAGTGACGAGCTTTTAACCAGGCAAAAAGGATCTTGGCGCGATCGTGCTTACAAACGATGAAGTCTTGCAACAATTTTGTGAG

>SMscf116-197061-197404

GTAGTGCTGGTCGCCAATTCTCTTCCCGCCCTGAACGACGTTACTGCAATGGAGCTACCTGAAATCGTTGCTGAGGCTGCTAAGCACTGTGACATTCTCCGAGGGGCGGCTGAAGCCGGGGGGCTGCTCGTGGATGCGATGATCAACGTCCAAGATAGCCCTAGAGAAAGCTCGTTGTCAGTACCGTTGATGGTGGTCGAAAACGGGTGTGGAAGTCCGTGCATAGACCTGCGACAAGTGAGCTCGGAGTTGGCTGCTGCTGCCAAGGATGCTGACCTG

>SMscf5077-22608-23239

ACAACTTTCAGCGCTCCAGAAGGAGACGAACCTCTGGCTTTGGATATGAATACCATGAGTAAAGGGTGGTTCAACGAGAAAAAGTGCCTGACCAACTGCGGCGAAGCTTCTCAGAGATGGTACCACGTCCCTCGTTCCTGGCTGTATCCATCTGGAAATCTGTTGGTCCTGTTTGAAGAATGGGGCGGGAATCCTTACGGGATCACTCTAGTGAAAAGGGAAGTAGCGAGTGTATGCTCTGATATATTTGAATGGCAACCAACCTTAGTGAACTGGCAGATGCTGTCATCAGGGAAAGTCGACAAACCATTGAGGCCGAAAGCTCACCTCTCGTGTGCTTCGGGTCAGAAGATCTCATCCATCAAGTTTGCCAGCTTTGGGACGCCTGAAGGCTCCTGTGGTAACTTCCGACAGGGGAGCTGCCACGCCTTCCATTCCTACGACGTTTTTGAGAGG

>SMscf550-26951-27637

AGATCTGGTCTCAGGGATCTCACAACAAGTAAAACTCCAGAAGCATCTATTTCTGCTGCTCTGTCAAGGGATACAAAACTGTTTGAGAGAACAGCTCCTTCAACTTATTGTGTACGCTCTCCTTATCGGAAGGATCCTGATGATGCAGAGGCGCTTCTCTCTGAAGCTAGGGAGAGAATTCGGGTGTATCAAAATGGAGATGTTGATGAAGAAGAACCAGATGTTGAAAAGGATGATCTTGAAAGAGATCAAGACTCTGAAAGTGATATTGGGGATGATCCTGATGTTGATGACTTGGATGCTTTGGCAAGTTAAAGGAATCTTCTCATTCCAGCGAAACAAGCAAATTTGAAGATATCTCTGGATATGGCAACGACAATTCCTGTAGTGAGTTGTTGGGAACTGCAATCTTGAAAAGCAGTTCAATGTTGACTGAATCAGCTAATGAGATGAAAGATAATGGAACAATGGCTGTCTCATGTGTCGATGCTTCTGGGGCTAACTCTCAGGTGTCCGCTTATGATCTTGAAGACAATGTAGTTGATGAATCTGGTCCTGGAGAACCATGGGTTCAGGGACTTACTGAAGGAGAGTATGCAGATCTCAGCACTGAAGAGCGTCTAAATGCTCTTGTTGCCTTGATTGGTGTAGCAAATGAAGGAAATGCAATCCGCGTCGCCTTGGAG

>SMscf2943-49422-49697

GTGGGGGATATATCAGGGTCTTTCCGTTGGGCTCCATTCCTGGCTTTGCAGAAAGCAAACTCGTTTTTGACAATGCTGTTGCTGCTATCCAAGTACAGGATGAAGAGCATCCCTGTAGTTGATCTGGGGGAAGCAAAGATGACAACATAATCACACAAAGCGCCGTTATTCACATGCTACAAGAGTGTGCTGGCCTCCACTGGTTCGAAAGCTGGGGTTCCAAAAAGCTATTTGAATTAGGCCTTCCATTGATGAAGGCCAGTAGCATGATCAAG

>SMscf432-13232-13866

GAGCAACCGAAATTACTCGTCGGAGACGGAAACGTACTGGGCATATATAGCTGAGGGAATGGGAGAGTTACAGAACTGGGAATCAGTTATGAAATATCAAAGAAGGAATGGATCTCTTTTCAACTGTCCCTCTACGACAGCAGCTGCTTTTATTGCCCTCCGCAATTCTGACTGCCTCAACTACCTGCATTTAGCCTTGAAGAAGTTTGGAAATGCAGTTTCTGCAGTTTATCCTCTAGATATATACTCTCAGTTGTGCACAGTTGACAATCTTGAAAGGTTGGGGATCAGCCAGTATTTTTCGACGGAGATTCAAAATGTGTTGGATGAAACGTACAGATGTTGGATGCAGGGCAATGAAGAGATATTCATGGACGCCTCAACTTGTGCTTTAGCATTCCGGACATTGCGATTGAATGGATATGATGTGACTTCAG
